# Freeze-derived heterogeneous structural color films

**DOI:** 10.1038/s41467-022-31717-2

**Published:** 2022-07-13

**Authors:** Shuangshuang Miao, Yu Wang, Lingyu Sun, Yuanjin Zhao

**Affiliations:** 1 Department of Clinical Laboratory, Nanjing Drum Tower Hospital, School of Biological Science and Medical Engineering, Southeast University, Nanjing, 210096 China; 2 Oujiang Laboratory (Zhejiang Lab for Regenerative Medicine, Vision and Brain Health); Wenzhou Institute, University of Chinese Academy of Sciences, Wenzhou, Zhejiang 325001 China

**Keywords:** Colloids, Displays, Nanoparticles

## Abstract

Structural colors have a demonstrated value in constructing various functional materials. Efforts in this area are devoted to developing stratagem for generating heterogeneous structurally colored materials with new architectures and functions. Here, inspired by icing process in nature and ice-templating technologies, we present freeze-derived heterogeneous structural color hydrogels with multiscale structural and functional features. We find that the space-occupying effect of ice crystals is helpful for tuning the distance of non-close-packed colloidal crystal nanoparticles, resulting in corresponding reflection wavelength shifts in the icing area. Thus, by effectively controlling the growth of ice crystals and photo-polymerizing them, structural color hydrogels with the desired structures and morphologies can be customized. Other than traditional monochromatic structure color hydrogels, the resultant hydrogels can be imparted with heterogeneous structured multi-compartment body and multi-color with designed patterns through varying the freezing area design. Based on these features, we have also explored the potential value of these heterotypic structural color hydrogels for information encryptions and decryptions by creating spatiotemporally controlled icing areas. We believe that these inverse ice-template structural color hydrogels will offer new routes for the construction and modulation of next generation smart materials with desired complex architectures.

## Abstract (web summary)

While most of the structural colored materials only present isotropic colors, limiting their functions and many practical applications, while realizing anisotropic structural color materials remains challenging. Here, the authors develop a freeze-derived heterogeneous structural color hydrogels for information encryption and decryption.

## Introduction

Structural color, resulting from the interaction of light with periodic nanoscale structures exhibiting varied refractive indices, has sparked widespread concerns by its prevalence in nature^1–7^. Inspired by natural creatures, various strategies have emerged for preparing artificial structurally colored materials with specific nanoarchitectures^8–13^. Among them, the self-assembled colloidal crystals (CCs) have offered unprecedented opportunities, because of their cheap cost, facile preparation procedures, the comparative simplicity of scaling up, and flexibility of building complicated nanometer-precision architectures^14,15^. In particular, the non-close-packed CCs formed by the charge repulsion of highly charged nanoparticles are attracting great interest for their relatively simple fabrication and flexible color adjustment^16^. Benefitting from these advantages, a variety of functional structurally colored materials have been fabricated for displaying^17^, anti-counterfeiting^18^, coding^19,20^, etc^21^. However, most of the structurally colored materials obtained in these traditional ways were with single nanostructures and could only present isotropic colors, which limits their functions and many practical applications^22–24^. Therefore, stratagem for generating anisotropic structurally colored materials with new architectures are still anticipated in various fields.

**Fig. 1:**
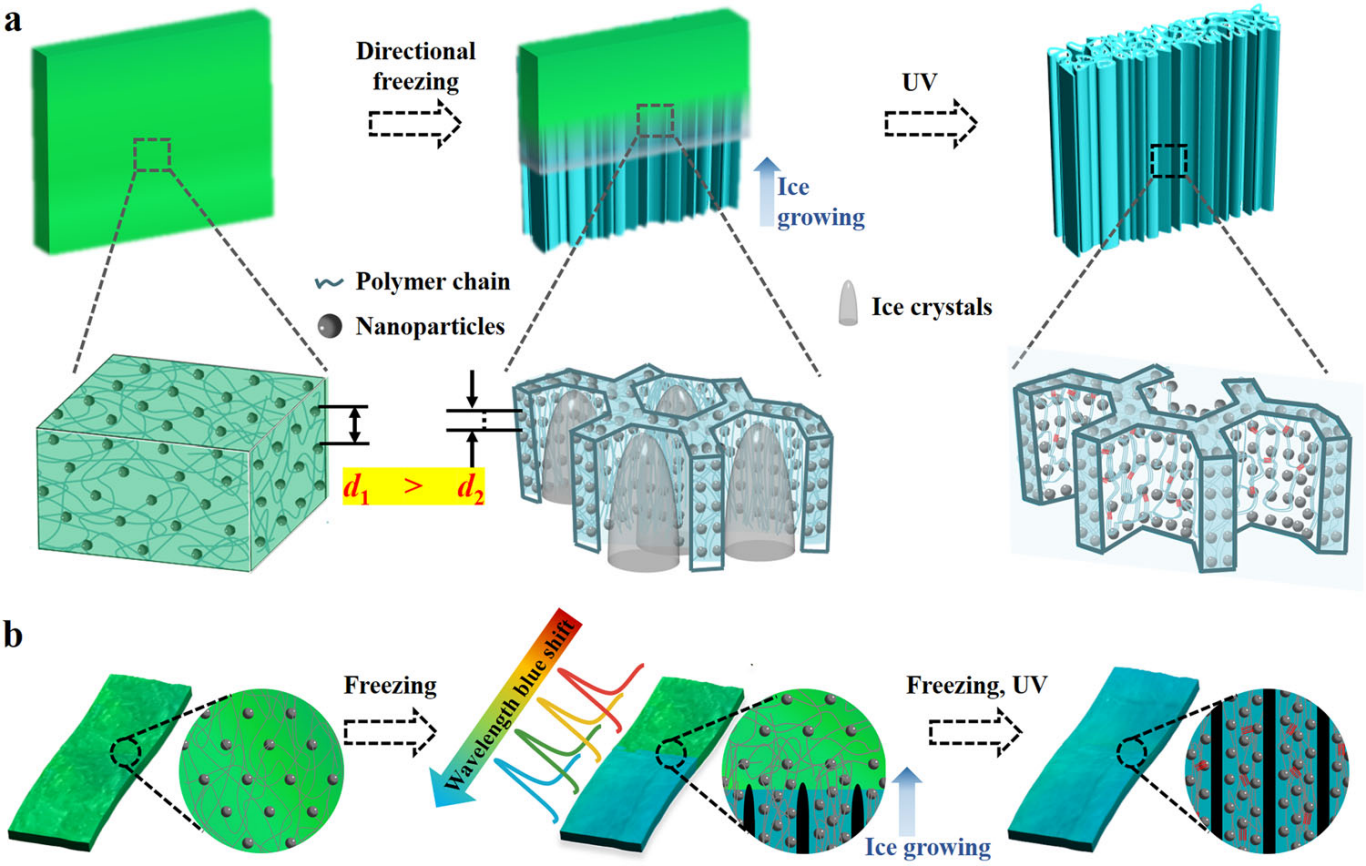
Principle of wavelength blue shift and fabrication route of ice-templated structural color films. **a** Schematic illustration of self-assembling nanoparticles based on ice-templating technology. **b** Scheme of the structural color film prepared by freeze-photopolymerization methods.

In this paper, inspired by the icing process in nature and freeze-casting technologies^25–28^, we proposed freeze-derived heterogeneous structural color hydrogel films with a series of distinctive features, as schemed in Fig. 1. Ice in nature has a micron-scale dendritic structure, and the unidirectional growth of ice crystals could cause the single orientation structure. With the structural characteristics, diverse building elements^29^, such as ceramics^30,31^, carbon nanotubes^32^, graphene^33,34^, polymers^35^, and boron nitride^36^, have been integrated into mass complex composites possessing nacre-like morphology via ice-templating^37–41^. In addition, benefitting from the porous nature of ice-template materials, the freeze-casting method is creatively used to prepare aerogel^42^, smart sponges^43,44^, polymeric woods^45^, and multifunctional cellular plastics^46^. Remarkably, strong tough hydrogels^47,48^ and fatigue-resistant hydrogels^49^, which are difficult to prepare by other methods, are successfully fabricated through the ice-template strategy^50^. However, the preparation of structural color hydrogel materials using freeze-casting methods has never been investigated.

Here, we carried out the desired structurally colored materials generation stratagem through a freeze-photopolymerization method. We found that the space-occupying effect of ice crystals was helpful for modulating the distance of non-close-packed nanoparticles, thus resulting in a corresponding blue shift of reflection wavelengths in the icing area. After being photo-polymerizing, the ice-template hydrogel with the same vivid structural color could be easily obtained. It was worth mentioning that compared with uniform ice growth, directional ice growth could further promote the assembly of colloidal nanoparticles through long-range orderly ice crystals arrangement and improve structural color performance on their surfaces. Based on these characteristics, heterogeneous structural color films with dual-color compartments were successfully fabricated, whose color could be flexibly adjusted by varying parameters such as nanoparticles solution concentration, subcooling, and water content in colloidal suspension. More attractively, the heterogeneous inverse ice-templated structural color films could be imparted with more complicated patterns via selectively controlling the freezing areas. Benefitting from the macroscopically visible color differences and spectral shift caused by icing, we demonstrated that the heterogeneous structural color films were valuable for information decryption. These features impart the inverse ice-template structural color hydrogels with great potentials in sensors, optical devices, anticounterfeiting, and so on.

## Results

### Fabrication of ice-templated structural color films

Freeze-casting (or ice-templating) can be employed as a method to construct sophisticated composites with desired structures. As shown in Supplementary Fig. 1, to observe the ice-template structures, the sodium alginate solution was slowly frozen and it can be seen clearly that local-orderly-oriented ice crystals were generated during the freezing process. Similarly, in polymer solutions, polymers will be trapped by the propelling water-ice interface. As schemed in Supplementary Fig. 2, the ice front topography of the frozen poly (ethylene glycol) diacrylate (PEGDA) aqueous solution showed an advancing dendritic geometry. Intriguingly, due to the gelation of PEGDA under the irradiation of ultraviolet (UV) light, the ice structure could be successfully replicated by the PEGDA hydrogel, which inspired us to utilize photopolymerizable hydrogels to replicate ice morphology.

**Fig. 2:**
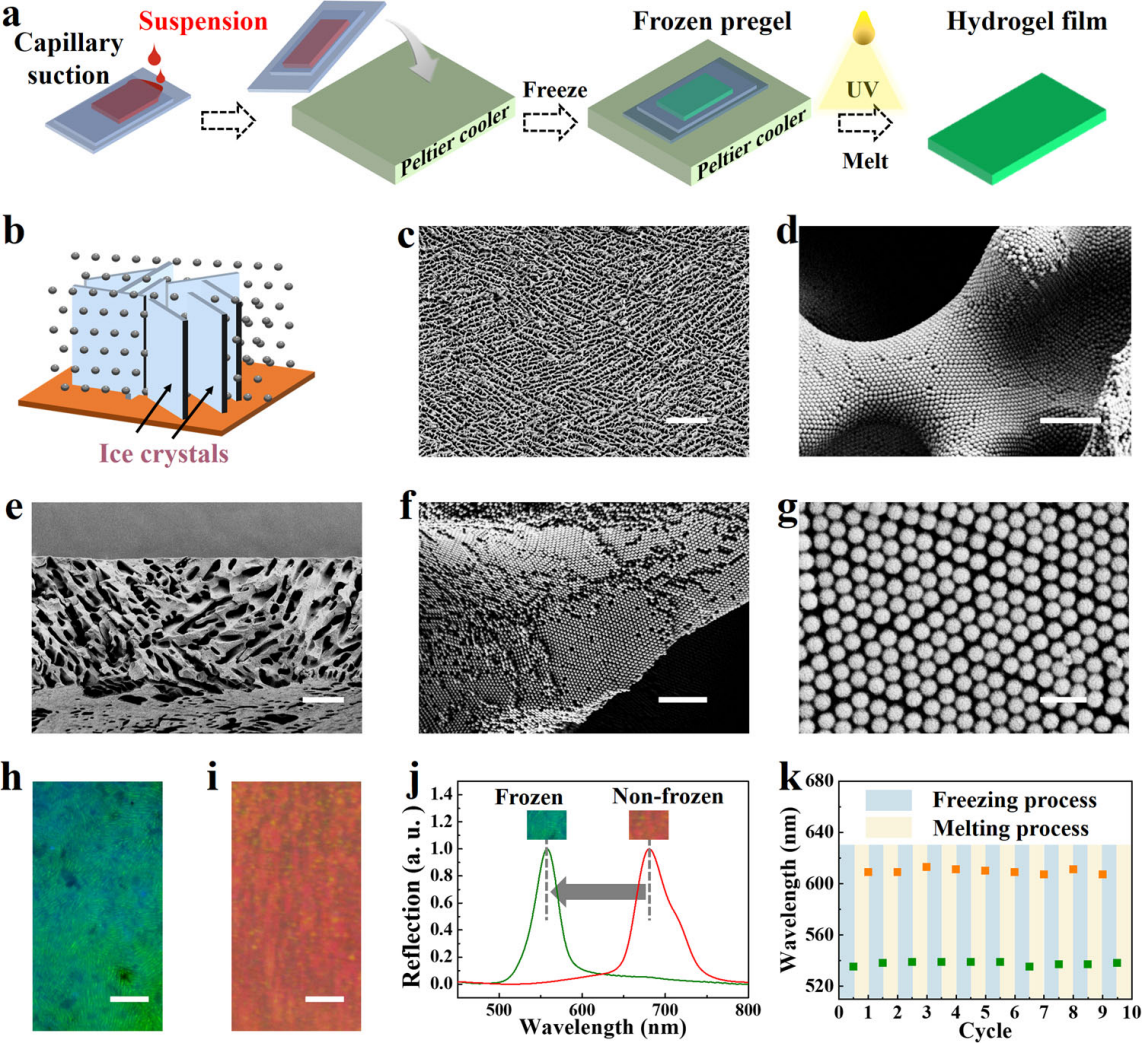
Optical and morphological characterization. **a** Schematic illustration of freezing pregel suspension on a uniform substrate. **b** Schematic illustration of nanoparticles assembly process based on ice-templating technology. **c**, **d** The surface of II-TSCHF. **e**–**g** The cross section of the film. **h**, **i** The optical images of structural color hydrogel films; (**h**) the II-TSCHF that was freeze-photopolymerized; (**i**) the hydrogel film that was photopolymerized directly without being frozen. The freezing temperature was −7.9 °C. The v/v ratio of H_2_O/PEGDA was 3:1. Nanoparticles with a diameter of 172 nm were employed. **j** Reflection spectra corresponding to the hydrogel films in (**h**, **i**). **k** The pregel suspension was cyclically frozen and melted at a constant temperature between −5.9 °C and ~20 °C. The v/v ratio of H_2_O/PEGDA was 1:1. Nanoparticles with a diameter of 172 nm were employed. The scale bars are 100 μm in (**c**, **e**), 2 μm in (**d**, **f**), 500 nm in (**g**), and 500 μm in (**h**, **i**).

In a typical experiment, we employed the ice-template to assemble non-close-packed colloidal crystal nanoparticles and adapted photopolymerization to fix the nanoparticle-assembled structure and replicate the ice-template structures, named the freeze-photopolymerization method. As illuminated by Fig. 2a, two cropped clean glass slides were prepared and then separated with spacers of a certain thickness to obtain the Hele-Shaw cell. The PEGDA pregel suspension containing surface-charged nanoparticles was slowly injected into the gap between the glass slides with a constant speed, in which case the nanoparticles entered the gap because of the capillary force. Subsequently, the Hele-Shaw cell containing the pregel suspension was put on the Peltier cooler with the subzero temperature, and the temperature of the pregel solution would drop slowly. After several minutes of subcooling, ice nucleation occurred, then ice crystals grew from the ice nucleus to the remaining region (Supplementary Fig. 3 and Movie 1). When the pregel suspension was completely frozen, the Hele-Shaw cell was immediately irradiated with UV light to gel and fix the microstructure generated by the ice crystals (Supplementary Fig. 4). After melting to remove ice-templates, the inverse ice-template structural color hydrogel film (II-TSCHF) was obtained.

The structure of II-TSCHF was characterized by the scanning electron microscopy (SEM) images, which presented a micro-nano hierarchical structure (Fig. 2b–g). For the II-TSCHF freeze-casted on the uniform subcooled Peltier surface, its surface was a long-range randomly but short-range orderly oriented micron-scale porous structure produced by the growing ice crystals, as shown in Fig. 2c and Supplementary Fig. 5. The size of this pore structure is approximately 1~100 μm. From the high magnification SEM image, it could be clearly seen that the pore wall was composed of silica nanoparticles assembled in an orderly manner (Figs. 2d, f). The reason why the stacking structure of SiO_2_ nanoparticles did not collapse was that the position of the nanoparticles was fixed by the gelatin of PEGDA. Additionally, due to the electrostatic repulsion effect, the highly charged nanoparticles presented a face-centered cubic non-close-packed arrangement (Fig. 2g), which was beneficial for modulating the structural color via changing the nanoparticles spacing. Due to the repulsion among the high charged nanoparticles, the characteristics of the particles packing were not side by side but with a certain distance. Because of the orderly arrangement of non-close-packed nanoparticles, the II-TSCHFs were endowed with vivid structural coloration (Fig. 2h). We found the color of II-TSCHF was apparently different from that of the hydrogel film that was photopolymerized directly without being frozen (Fig. 2i). The corresponding spectra of the films were also measured, displaying photonic bandgap feature (Fig. 2j). The photonic bandgap, derived from non-close-packed crystal micro-structures of highly charged nanoparticles, was able to regulate light and reflect certain wavelengths. The main wavelength position ***λ*** of the reflection peak of the II-TSCHF can be calculated with the help of Bragg’s equation

1 $$\lambda =2d{n}_{{{{{{\rm{ave}}}}}}}$$

wherein *d* refers to the (111) diffractive layers’ spacing, *n*_ave_ denotes the average refraction index. In addition, when freezing–melting cycles were performed at a constant freezing temperature for the unpolymerized pregel suspension, the dynamic reflective peak also cycled between ~535 nm and ~610 nm (Fig. 2k), indicating its stable optical property.

### Designability, tunability, and applicability

**Fig. 3:**
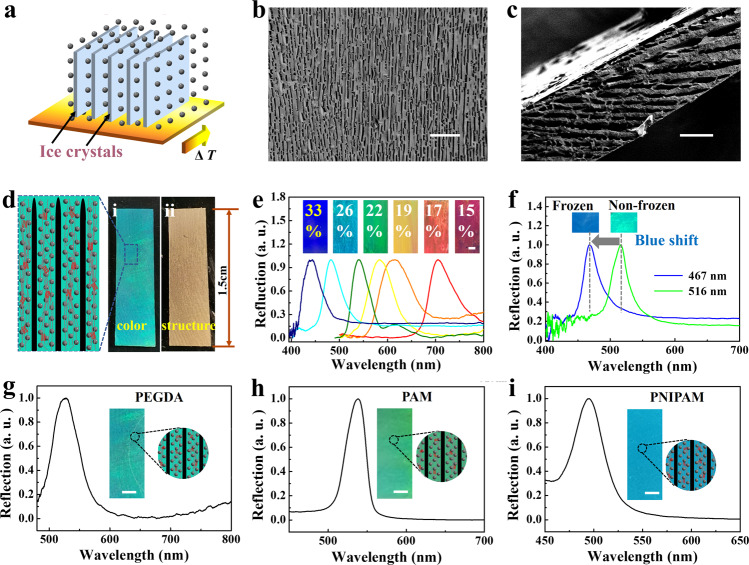
Designable micrometer-sized structure, tunable structural color, and wide-applicable photopolymerizable hydrogel materials. **a** Schematic illustration of nanoparticles assembly process based on unidirectional ice-templating technology. **b**, **c** The SEM images of the surface (**b**) and cross section (**c**) of a directional II-TSCHF. **d** Optical images of the directional II-TSCHF; (i) the reflection image showed the color; (ii) the opaque transmission image displayed the directional stripe caused by the ice-template structure. **e** Optical images and corresponding reflection spectra of II-TSCHFs with various nanoparticles solution concentration. **f** Wavelength variations of the directional frozen II-TSCHF and the non-frozen film. The subcooling is 7.9 K. The v/v ratio of H_2_O/PEGDA was 1:1. Nanoparticles with a diameter of 172 nm were employed. **g**–**i** Optical images and corresponding wavelength peak of the directional II-TSCHF fabricated by various hydrogel materials; **g** PEGDA; **h** PAM; **i** PNIPAM. Nanoparticles with a diameter of 172 nm were employed. The temperature was roughly −7.9°C. The v/v ratio of H_2_O/PEGDA was 1:1. Scale bars are 200 μm in (**b**), 100 μm in (**c**), 2 mm in (**e**), and 1 mm in (**g, h, i**).

The micrometer-level architectures of II-SCHFs are templated by the ice crystal, endowing the II-SCHFs with designable micrometer-sized structure features. Especially, the porous lamellar structures are successfully constructed in structural colors materials. The growing ice displays strong anisotropic growth kinetics, enabling the possibility of shaping ice flakes with a very high aspect ratio under steady-state temperature gradient conditions. Directional structure in II-SCHFs can be achieved by oriented aligned ice platelets. Taking advantage of traveling temperature gradients, the unidirectional ice platelets growth was achieved after ice nucleation (Supplementary Fig. 6). Under the synergistic effect of ice fronts squeezing and electrical force, the highly charged nanoparticles self-assembled into an orderly micro-nano structure, displaying vivid structural color. It was evident that, as the freezing front propelled, the freezing area gradually became green color, indicating excellent self-assembly (Supplementary Fig. 7). Similarly, through replicating the unidirectional ice-template structures and UV solidifying (Supplementary Fig. 8), the free-standing II-TSCHF with a directionally oriented structure was fabricated (Fig. 3a–c, Supplementary Figs. 9 and 10). The directional ice-templated film displayed a centimeter-scale long-range-oriented arrangement of pore structures similar to that in nacre. Combining well-ordered micrometer-scale structures with nanometer-scale non-close-packed particles, the directional ice-templated film enabled the bright structural color feature (Fig. 3d).

The structural color of II-TSCHFs was tunable via non-close-packed nanoparticles solution concentrations. According to Eq.(1), as the materials keep constant, *λ* is related with *d*_111_. Precisely, *d*_111_ is dependent on the distance of highly charged silica nanoparticles, which determines the structural color of II-TSCHFs. To demonstrate that the structural color of the II-TSCHF could cover the entire visible spectrum, the PEGDA solution (50 v/v %) was prepared. Subsequently, by mixing a constant volume PEGDA solution with divergent amounts of surface-charged nanoparticles, the colloidal suspension with various nanoparticles solution concentration was obtained. After freezing and UV light solidifying, we obtained the II-TSCHFs of various colors, and the reflection spectra were measured correspondingly, as shown in Fig. 3e. Remarkably, the liquid solution content in suspension should be controlled in a specific range. If the liquid content in colloidal suspension is greatly low, the film color will reach the UV spectrum region, appearing white or white mixed with blue-violet (Supplementary Fig. 11). Correspondingly, if the liquid content was raised too much, the film color would reach the infrared spectrum region, appearing white or white mixed with red. In the process of preparing ice-templated structure color films of various colors, we found that their colors were significantly different from that of the non-frozen structural color film. By measuring their reflection spectra, we found that the reflection peak of the ice-templated hydrogel was blue-shifted compared to the spectrum of non-frozen ones (Fig. 3f).

Besides, the presented freeze-photopolymerization method was generalizable to a wide range of hydrogels. The hydrogels that satisfy the following two conditions were applicable, which were that surface-charged nanoparticles were able to disperse into the solution to produce a colloidal suspension with vivid structural color and the hydrogels were photopolymerizable, respectively. To verify this hypothesis, besides PEGDA hydrogels (Fig. 3g), we also selected the most commonly used poly(acrylamide) (PAM) hydrogel. Adopting the same fabrication method as mentioned above, the PAM II-TSCHF was successfully fabricated, displaying bright structural color (Fig. 3h). Its micrometer-level pore structure showed that the ice crystal structure had been successfully replicated, validating the micro-nano hierarchical structural features of II-TSCHFs (Supplementary Fig. 12a). Furthermore, for stimulus-responsive hydrogels, such as poly(N-isopropylacrylamide) (PNIPAM), we also demonstrated its feasibility in the construction of the II-TSCHFs (Fig. 3i and Supplementary Fig. 12b). All these results indicated that the II-TSCHFs were generally applicable towards a wide range of photopolymerizable hydrogels. In addition, although the role of the hydrogel in this paper is mainly to fix the frozen structure, resulting in that the structure caused by the ice template can be retained after the ice melts, we surmise the matrix would affect the freezing process and colors, which provides a critical research direction of our future study. We believe our works will bring inspiration to a variety of application fields.

### Wavelength blueshift induced by freezing

**Fig. 4:**
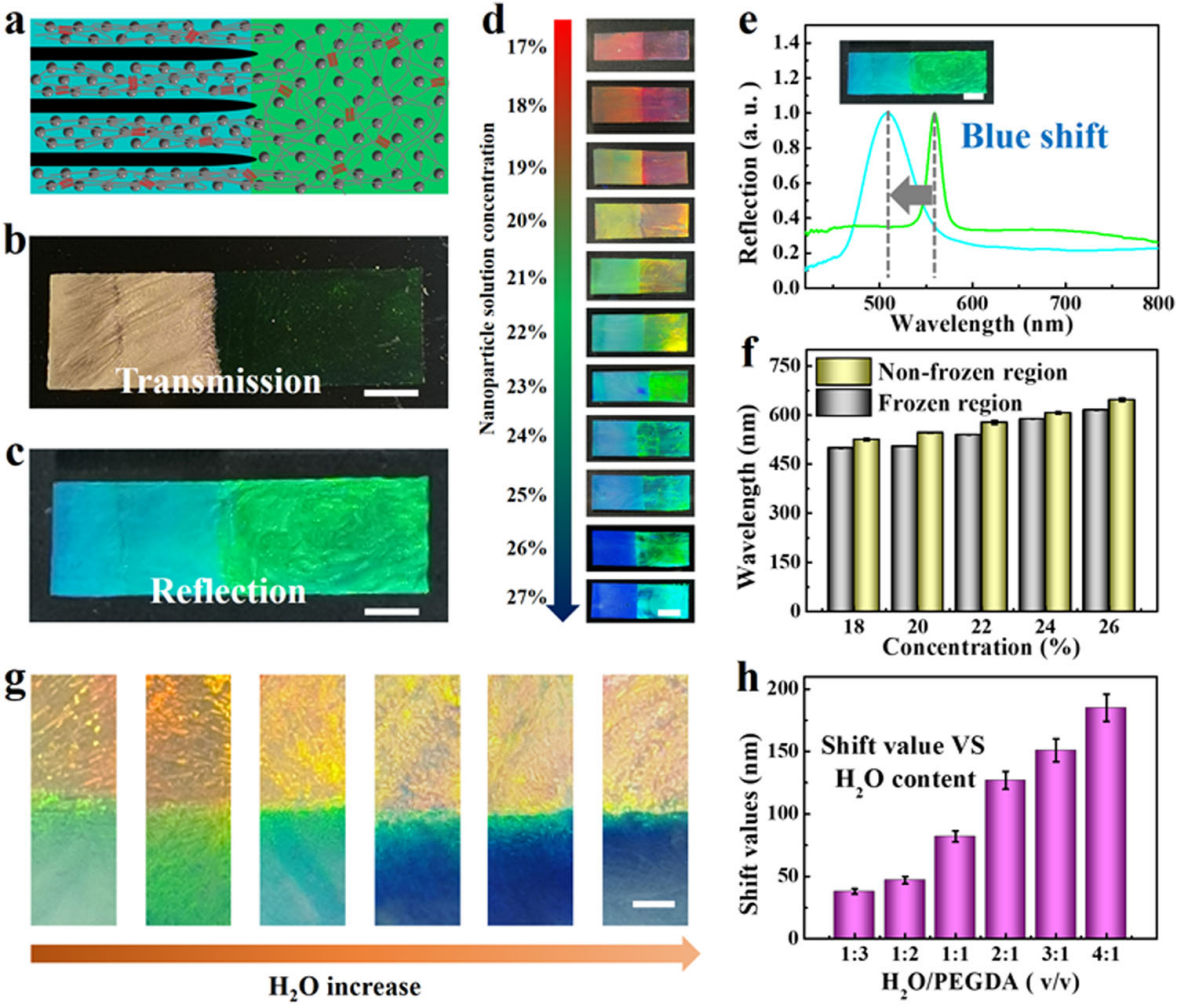
Wavelength blue-shift induced by freezing. **a** Schematic illustration of dual-color ice-templated film. **b**, **c** The transmission image (**b**) and reflection image (**c**) of a dual color heterogeneous II-TSCHF. The right part is nonfrozen zone and the left part is frozen zone. The subcooling is 7.9 K. The v/v ratio of H_2_O/PEGDA was 1:1. A pregel suspension with nanoparticles solution concentration of 23 % was used. Nanoparticles with a diameter of 145 nm were employed. **d** Optical images of dual color heterogeneous II-TSCHF with different nanoparticles solution concentration. The v/v ratio of H_2_O/PEGDA was 1:2. The subcooling is 9.2 K. Nanoparticles with a diameter of 145 nm were employed. **e** Reflection spectrum of the heterogeneous structural color hydrogel corresponding to (**c**). **f** Reflection peak wavelengths of various dual color heterogeneous structural color hydrogels with different nanoparticle solution concentration corresponding to (**d**). **g** Optical images of the dual color heterogeneous hydrogel films of different liquid water content in colloidal suspensions. The upper part is nonfrozen zone and the lower part is frozen zone. The subcooling is 13.4 K. A suspension with nanoparticles solution concentration of 20% was used. **h** Relationships between shift values of heterogeneous structural color films and different liquid water content in colloidal suspensions, in which case the subcooling is 13.4 K. The error bars represent the standard deviations derived from at least three specimens. Scale bars are 2 mm in (**b**–**e**), 1 mm in (**g**).

In order to clarify the spectrum difference between the frozen and non-frozen structure color film, we proposed a mechanism interpretation, named icing blue shift. There was a liquid water phase that could be frozen and a non-aqueous phase (nanoparticles and PEGDA) that could not be frozen in the colloidal suspension. On the low-temperature cold substrate, when the nucleation energy barrier was reached, the liquid water in the suspension firstly nucleated to form an ice embryo. Next, ice grew from the embryo, free liquid water molecules near the ice embryo were captured and fixed at the ice-water interface, and a liquid-solid phase transition occurred. Simultaneously, ice crystals squeezed out the non-aqueous phase. Because of the space-occupying effect of ice crystals, the distance between nanoparticles was reduced. Corresponding reflection spectra of obtained II-TSCHFs would be blue-shifted according to Bragg Eq.(1), as schemed in Fig. 4a.

In order to verify the icing blue shift principle proposed above and reveal the influence of icing behavior on the reflection spectrum of the structured color film, we designed a heterogeneous structural color film, in which the half area was frozen and the rest kept non-frozen (Supplementary Figs. 13, 14, and Movie 2). To be specific, we suspended half of the Hele-Shaw cell and placed the other half onto the Peltier cooler, the temperature of which was subzero. After slow freezing, UV irradiation, and removal of ice-templates, the final heterogeneous hydrogel film with two colors was obtained. From the transmission and reflection optical photography of a dual-color heterogeneous hydrogel film, it could be found that the transmission photograph was observed that the half area in the frozen area became opaque because of the porous structure left by removing ice crystals, while the half film in the non-frozen area appeared more transparent (Fig. 4b). In addition, the structural color of the non-frozen area was green, while the structural color of the frozen area was cyan, which was consistent with the proposed principle (Fig. 4c). The SEM images more intuitively showed the microstructure of the dual color film, elucidating the underlying physics of icing blue shift. As schemed in Supplementary Figs. 15–17, the frozen area on the left had micro-porous structure, while the nonfrozen area on the right showed flat surfaces. From the high magnification SEM image, it can be seen that ice crystals with a scale characteristic of tens of microns have produced a micron-scale porous structure in the film, validating the icing blue shift caused by the squeezing of ice-templates. Furthermore, by varying the concentration of nanoparticles solution, various heterogeneous hydrogel films with dual color compartments were fabricated (Fig. 4d). The reflection spectra of the frozen zone and non-frozen zone in dual-color heterogeneous structural color films displayed apparently distinction (Figs. 4e, f and Supplementary Fig. 18), demonstrating the squeezing effect of ice-templates on high charged nanoparticles would cause blue shifts of the reflection peak wavelengths. In addition, to reveal the optical properties of the whole surface of the heterogeneous hydrogel films, we measured and analyzed the spectra of 138 array sites (6 rows × 23 columns) on the surface. The results indicated that the frozen and nonfrozen regions displayed a distinct photonic band gap difference (Supplementary Fig. 19). All these results illustrated that the structural color films possessed heterogeneous feature, which originated from the distinctive structure of the films composed of frozen area and non-frozen area, as well as heterogeneous properties, including two photonic band gaps (two colors) and two structural morphologies. Such heterogeneous structural color films with a facile and flexible advantage in material preparation open up new avenue for color patterning and information safety, showing great potentials in sensors, optical devices, anticounterfeiting, color imaging technology and so on.

The relation between water content and wavelength blue shift was researched. As schemed in Supplementary Fig. 20, the water component in the colloidal suspension would transform to ice-templates. After removing ice crystals templates, the pores remained. Therefore, the water content in the colloidal suspension corresponds to the pore size and may affect the icing blue shift. To verify this surmise, we uniformly dispersed the highly charged colloidal nanoparticles in the prepared pre-solution. The pre-solutions were composed of water and PEGDA in different volume ratios, including 1:3, 1:2, 1:1, 2:1, 3:1, and 4:1 (H_2_O: PEGDA), respectively. The same weight of nanoparticles was dispersed into the same volume of pre-solutions with different H_2_O/PEGDA volume ratios to obtain orange colloidal crystals suspensions L_1_~L_6_. The dual color heterogeneous structural color films F_1_~F_6_ were prepared by applying freeze-photopolymerization technology to the six groups of orange suspensions L_1_~L_6_, as shown in Fig. 4g. The color of frozen area, in turn, varied from green to blue with H_2_O content increasing. This result showed that as the H_2_O content in the pre-solution increased, the value of icing blue shifts gradually increased (Fig. 4h and Supplementary Fig. 21). Besides, in order to investigate the molecular weight of PEGDA on freezing, PEGDA of various molecular weight (200, 400, 700, or 1000 Da) and H_2_O were mixed at the v/v ratio of 1:1. It was found that the PEGDA-200 was insoluble in water and phase separation occurred (Supplementary Figs. 22a and b), indicating that it cannot be used for freeze-casting to fabricate inverse ice-template structural color hydrogels. In contrast, PEGDA-400, PEGDA-700, and PEGDA-1000 were all capable of forming transparent aqueous dispersions. Therefore, PEGDA-400, PEGDA-700, and PEGDA-1000 were adopted to test the effect of the molecular weight on freezing and the wavelength blue shift. It was shown that as molecule weight increased, freezing became more difficult and the wavelength blue shift decreased under the identical other conditions (Supplementary Fig. 22c).

**Fig. 5:**
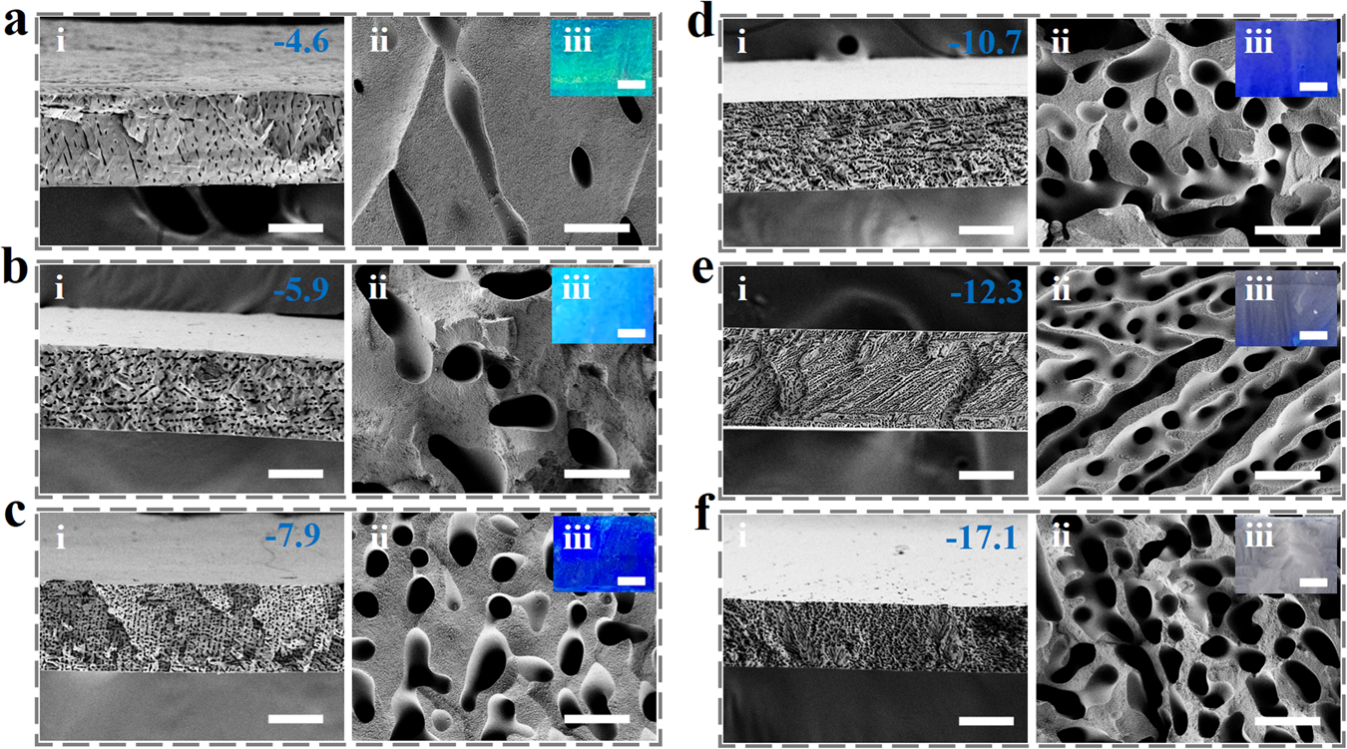
Comparison of ice-templated structural color hydrogels under different freezing temperatures. The temperatures are −4.6 °C in (**a**), −5.9 °C in (**b**), −7.9 °C in (**c**), −10.7 °C in (**d**), −12.3 °C in (**e**), and −17.1 °C in (**f**). The v/v ratio of H_2_O/PEGDA was 1:1. Nanoparticles with a diameter of 145 nm were employed. Scale bars are 100 μm in (i), 10 μm in (ii), 2 mm in (iii).

The effects of subcooling and freezing time on the icing spectrum blue shift were analyzed. It was demonstrated that the icing blue shift value raised with the increase of subcooling within a certain temperature range (Supplementary Movies 3) and the process of color changes was macroscopically reversible for specific freezing temperature (Supplementary Movies 4). However, when the freezing temperature continued reducing, the structural color faded or even disappeared (Supplementary Figs. 23 and 24). When the subcooling is low, the ice crystals grew slowly and ice branches are relatively few and loose (Fig. 5a–c), in which case the ordered nanoparticles in suspension rearranged into another ordered structure under the squeezing effect of the ice crystals, resulting in vivid color and photonic bandgap (Supplementary Fig. 25). In contrast, for the case of high subcooling, the ice crystals grew rapidly and densely, the blue-shift increased while reflection intensity weakened (Fig. 5d–e and Supplementary Fig. 25), the probable explanation for the disappearance of color is that the distance between nanoparticles was greatly reduced and the reflection peak has entered the ultraviolet spectrum range (Supplementary Fig. 25, 28a, and 11). According to our experiments, the temperature range for producing vivid structural color is roughly from -10 °C to 0 °C. Nonetheless, this does not imply that the higher temperature, the better experimental settings. We have to note that ice nucleation requires a certain degree of subcooling to overcome the nucleation energy barrier, resulting that the suspension will not freeze at relatively higher temperatures (such as −2 °C). Therefore, appropriate low subcooling is a critical condition for obtaining desired structural color. For our tests, the suggested temperature is approximately -5.9 °C and corresponding freeze time is ~10 min.

In addition, for the dual color heterogeneous structural color hydrogel films, we found that after half of the colloidal suspension on the Peltier surface has completely frozen, a transition zone appeared around the solid-liquid interface as freezing time further went by (Supplementary Figs. 26 and 27). The reason was that there is a temperature gradient between the edge of the subzero surface and the surrounding ambient air, causing the structural color gradient at the boundary of the Peltier cooler (Supplementary Fig. 28). Based on the above analysis, the color of II-TSCHFs can be slightly adjusted by modulating subcooling and freezing time. Notably, the influence of the water content in the colloidal suspension on blue shift is primary, subcooling and freezing time can be used to fine-tune the structure color in a small range.

### Freeze-assisted patterning strategy and freeze-decrypted method

**Fig. 6:**
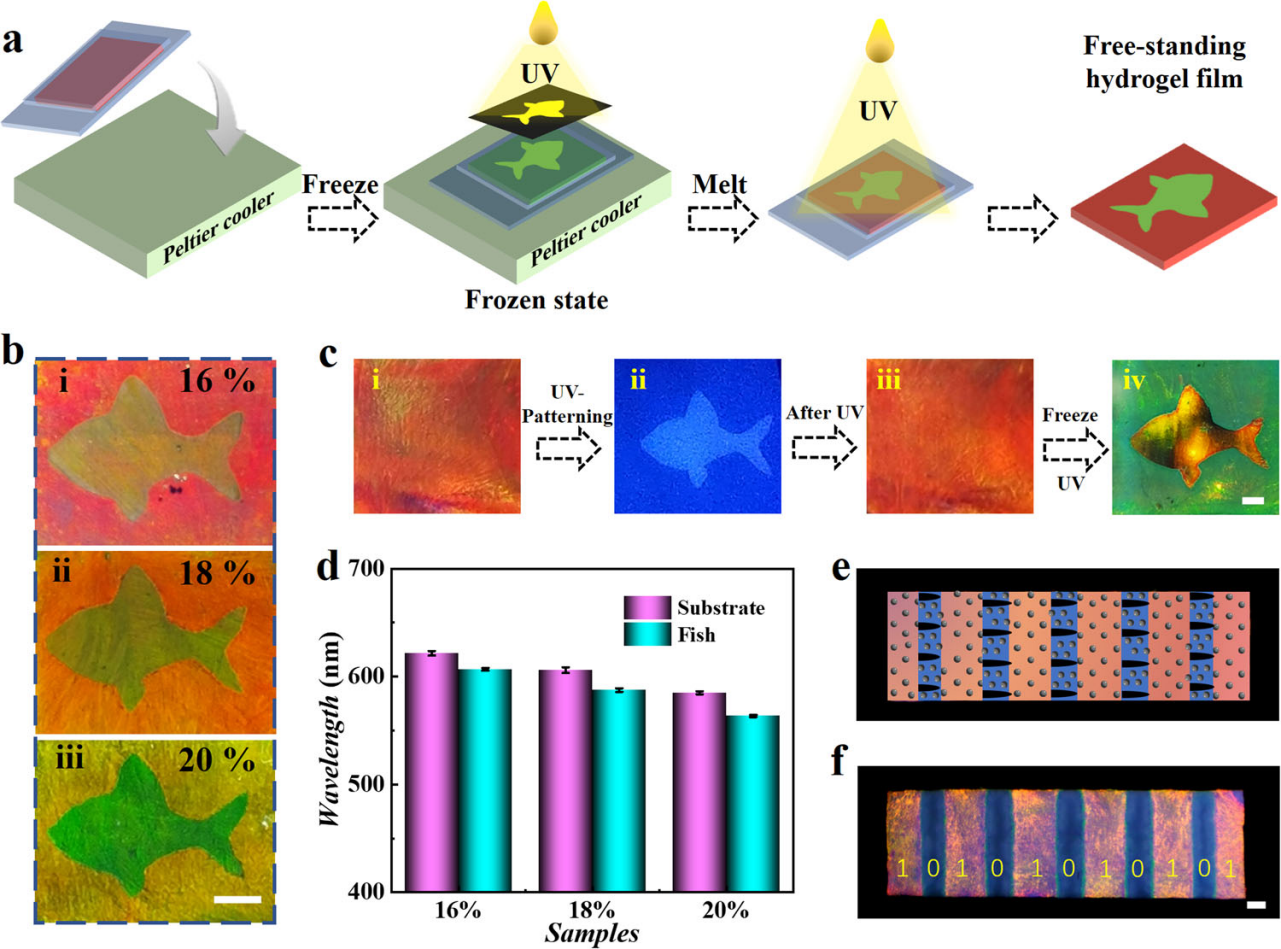
Heterogeneous structural color hydrogels with designed freezing geometries. **a** Schematic illustration of preparing heterogeneous structural color hydrogels possessing dual-color compartments. **b**, **d** Optical images (**b**) and corresponding reflection peak wavelengths (**d**) of patterned structural color hydrogel with various nanoparticles solution concentration. **c** (i-iii) The suspension film was spatially irradiated by UV light via masks to construct a fish pattern; (iv) the hidden pattern was displayed via freeze-photopolymerization method. **e, f** Schematic illustration (**e**) and optical images (**f**) of structural color hydrogels with bar coding geometry. Binary code: 10101010101^2^ = 1365^10^. The error bars represent the standard deviations calculated from three specimens. Scale bars are 2 mm in (**b**), 1 mm in (**c**), and 500 μm in (**f**).

Patternable materials are highly demanded in anti-counterfeiting and graphic displays applications. With the mechanism of freeze-induced wavelength blue shift, we proposed a strategy to construct spatiotemporally controllable geometry patterns on structural color hydrogel films. As shown in Fig. 6a and Supplementary Movie 5, the suspension was firstly frozen on the cold Peltier surface, accompanied by icing blue shift. Under frozen state, the suspension film was spatially irradiated and the irradiated area was photocured. Subsequently returning to room temperature, the color of the non-irradiated region returned to its original color, while the irradiated region nearly kept the color of frozen state. After UV-photocuring the remaining region, we successfully prepared a free-standing film with a fish pattern. By varying nanoparticles solution concentration, patterned hydrogels with various colors were fabricated (Fig. 6b). Reflection peak wavelengths of the patterned area and the substrate were measured (Fig. 6d), suggesting distinguish wavelength difference. Therefore, spatially selective photopolymerization at non-frozen state and frozen state can build heterogeneous structural color hydrogel membranes with designed patterns.

Reversely, we changed the order of photopolymerization to pattern the structural color hydrogel (Supplementary Fig. 29). The suspension film was firstly spatially irradiated at ambient temperature and the radiated region was cured, while the difference between the irradiated and non-irradiated was almost invisible (Fig. 6 ci–iii). Then suspension was directly frozen at low temperature and the hidden pattern appeared due to mismatch between the irradiated and non-irradiated regions during the freezing process (Supplementary Movie 6). After polymerizing the entire film in a frozen state, the patterned heterogeneous hydrogel films were also fabricated (Fig. 6 civ). In addition, taking advantage of the color difference between frozen and unfrozen, we demonstrated that this material can be applied to color binary coding (Fig. 6e, f), which provided a convenient and cost-efficient way of the commercial barcode. In our color patterning tests, the achieved minimum size was ~500 μm, which was constrained by our photomask employed. It was anticipated that if the size of the photomask was reduced, the resolution of the color patterning would improve further. Such a facile patterning method based on ice-templated structural color hydrogels made it possible to achieve diversified microscale structures and various colors on a single hydrogel film, which was difficult to realize by existing patterning methods. Besides, the structurally colored materials generated by the freezing method no longer rely on the cooling system after photopolymerization. Based on this technology, a range of structurally colored materials or heterogeneous materials can be produced, which would provide many ideal candidates for next-generation smart materials. These features suggested that freeze-photopolymerization technique, as a supplement to other fabrication processes for the manufacture of structurally colored materials, displays a facile and flexible advantage in material preparation.

**Fig. 7:**
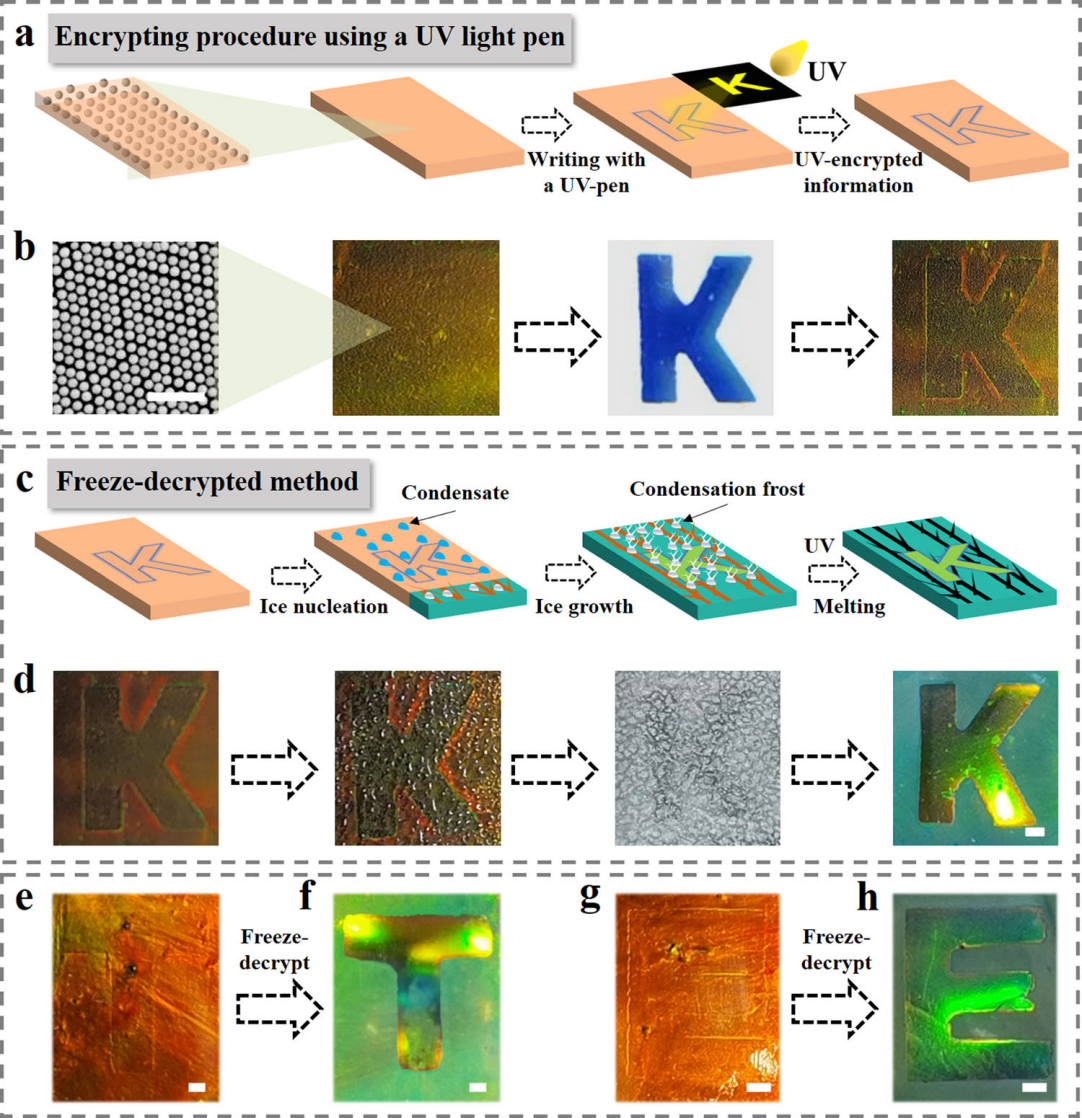
A proof-of-concept demonstration of information encryption and decryption technology via icing wavelength blue shift. **a, b** Schematic illustration (**a**) and experimental images (**b**) of encryption process through UV light, in which an encrypted letter “K” was written onto the pregel film using a UV light pen with the help of photomask. **c**, **d** Illustration (**c**) and optical images (**d**) of decryption process via freeze-photopolymerization. **e**–**h** Optical images of structural color hydrogel with letter information, including the UV-encrypted information (**e, g**) and the freeze-decrypted results (**f, h**). Scale bars are 1 μm in (**b**), and 1 mm in (**d**–**h**).

Furthermore, combining heterogeneous ice-templated structural color hydrogel films and UV-patterning features, we proposed an information encryption and decryption method. As schemed in Figs. 7a, b, UV light was employed to write a hidden letter K, and the irradiating-patterned region and the remaining region displayed no difference in photonic bandgap property (Supplementary Fig. 30), suggesting that the information encryption was successfully achieved. To display the hidden message, the information carrier was frozen to decode encrypted information. Under the squeezing of propelled ice-templates, nanoparticles of the unirradiated area were compressed, resulting in a color distinction between the letter information and the information carrier (Fig. 7c, d). Besides, a series of other letters were successfully encrypted via UV light pen and decrypted via freeze-photopolymerization method (Fig. 7e–h and Supplementary Fig. 31). In addition, it was demonstrated that the angle dependence of II-TSCHFs was greatly weakened after freezing (Supplementary Fig. 32), although there was still a minor shift for large inclination, which may open up new avenues for the design and application of angle-independent structurally colored materials. These features impart the II-TSCHFs with great potentials in sensors, optical devices, anticounterfeiting, and so on.

## Discussion

In summary, inspired by the freezing process and ice-templating technology, we have adapted a freeze-photopolymerization technique to construct inverse ice-template structural color hydrogel materials with micro-nano multiscale structures. The materials have excellent designable micro-sized morphological and facile structural color modulation features. Besides, due to the squeezing effect of growing ice crystals, spacings between surface-charged nanoparticles decreased, resulting in a wavelength blue shift. According to the icing blue shift mechanism, we proposed a freeze-patterning strategy and information encryption and decryption method, making II-TSCHFs a promising material for biomedical analysis, sensors, optical devices, information safety, and anticounterfeiting.

## Methods

### Materials

Highly-charged SiO_2_ nanoparticles, the diameters of which were 145, 172, 190 nm, were self-fabricated. Poly (ethylene glycol) diacrylate (PEGDA, M.W. 200, 400, 700, 1000), 2-hydroxy-2-methyl-1-phenyl-1-propanone (HMPP), acrylamide (AM), N-isopropylacrylamide (NIPAM), and N, N’-methylenebisacrylamide (BIS) were acquired from Sigma Aldrich (St. Louis, MO). Unless otherwise specified, PEGDA (M.W. 700) was employed.

### Preparation of the colloidal suspension

The colloidal suspension was composed of the PEGDA aqueous solution and surface-charged SiO_2_ nanoparticles. Firstly, PEGDA and H_2_O were mixed at the v/v ratio of 1:1, followed by adding the HMPP (1 v/v %) to the solution. After adequately mixing them, the PEGDA aqueous solution was obtained. Next, the highly-charged SiO_2_ nanoparticles were dispersed into the prepared PEGDA solution until the final nanoparticle concentration was 20 wt %. After ultrasound treatment for 30 min and vortex oscillation for 10 min, the colloidal suspension with vivid structural color was acquired. Remarkably, when the silica nanoparticles are completely dispersed in the PEGDA hydrogel precursor solution, an appropriate amount of ion exchange resin should be added to adsorb the cations in the system and ensure the stability of the self-assembled structure of the silica nanoparticles. Besides, by varying the amounts of nanoparticles added to the PEGDA solution, the pregel suspensions of various nanoparticles solution concentration (15–33%) were prepared. Unless otherwise specified, nanoparticles with a diameter of 145 nm were employed.

### Fabrication of the II-TSCHF

The II-TSCHF was fabricated based on freeze-photopolymerization methods. Two clean, hydrophilic slides were separated by spacers with a certain thickness to obtain a Hele–Shaw cell. The pregel was injected uniformly and slowly into the gap between the two slides. Then the Hele-Shaw cell was moved to a low-temperature Peltier cooler. After the water in the suspension is completely frozen, the Hele–Shaw cell was polymerized under UV light exposure for approximately 10 s with an intensity of 500 mW/cm^2^ to replicate the ice-crystals structure. Finally, the II-TSCHF with randomly oriented pore structures was obtained after removing ice-template at room temperature. Unless otherwise specified, UV light was irradiated for 10 s at an intensity of 500 mW/cm^2^ for all experiments.

### Directional ice growth via constant velocity

The Hele-Shaw cell containing nanoparticles pregel suspension was placed on the Peltier cooler with a thin ethanol liquid film separating them. The syringe pump (Longer Pump LSP01) was used to push the Hele–Shaw cell structure at a constant speed, so as to realize a fixed freezing speed of the colloidal suspension along the velocity direction. After the suspension was completely frozen, UV light was used to solidify it. Thus, the directional II-TSCHF was fabricated.

### The verification of wavelength blue-shift induced by freeze-casting

We fabricated heterogeneous structural color films with half-frozen area and half non-frozen area to validate the icing blue-shift mechanism. The Hele-Shaw cell with nanoparticle suspension was prepared based on the above method. A tiny part of the Hele-Shaw cell (denoted as A) was directly in contact with the square Peltier cooler platform, the temperature of which was set relatively low, while the rest (denoted as B) was suspended in the air without contact with the cold platform. After ice nucleation occurred in part A, a half (denoted as C) of the Hele-Shaw cell was pushed onto the Peltier cooler, simultaneously the temperature of Peltier cooler was set to the specified value. Subsequently, ice crystals grew from Part A to the rest of Part C. When Part C was completely frozen, the suspension was immediately polymerized under UV light exposure, the dual-color heterogeneous structural color film with half-frozen area and half non-frozen area was finally formed.

### The construction of patterned heterogeneous structural color hydrogel films

Spatially defined patterns are achieved via masked UV irradiations at frozen state and molten state. With the help of embossing molds, different patterned masks were prepared. The Hele-Shaw cell containing the suspension was put on the Peltier cooler to freeze at a specific temperature, and then we used masks to selectively photopolymerize the pregels, UV light was irradiated for 2 s with an intensity of 500 mW/cm^2^. Subsequently, the temperature was raised to remove the ice templates, followed by photopolymerizing and the rest, thus patterned heterogeneous hydrogel films were obtained. Similarly, the suspension film was firstly spatially irradiated (UV light was irradiated for 2 s with an intensity of 500 mW/cm^2^) at ambient temperature via masks and then directly frozen at low temperature. Subsequently, the entire film was polymerized in a frozen state (UV light was irradiated for 10 s at an intensity of 500 mW/cm^2^), and the patterned hydrogel films were also fabricated.

### Characterization

Reflection spectra were acquired at a constant glancing angle employing an optical microscope equipped with a fiber-optic spectrometer (Ocean Optics, USB2000-FLG). The spectra of the structural color hydrogels were measured before water volatilization. The light beam size for the reflectance measurement is ~0.636 mm^2^. SEM images were obtained by scanning electron microscopy (SEM, Hitachi S-3000N). Optical images were taken using an optical microscope (Olympus BX51) equipped with a CCD camera (Media Cybernetics Evolution MP5.0) and a digital camera (Canon5D Mark II, Japan).

## Supplementary information

Supplementary Information

Description of Additional Supplementary Files

Supplementary Movie 1

Supplementary Movie 2

Supplementary Movie 3

Supplementary Movie 4

Supplementary Movie 5

Supplementary Movie 6

## Data Availability

All SEM and spectra data supporting the findings of this study are provided in the paper and Supplementary Information files. All other relevant data are available from the corresponding author (Y. Z.) on request.
