# Supplementary material for: Freeze-derived heterogeneous structural color films
Source: Nat Commun. 2022 Jul 13;13:4044. doi: 10.1038/s41467-022-31717-2 (PMC9279407; doi:10.1038/s41467-022-31717-2)
Supplement: Supplementary file 1 — Supplementary Information [file 41467_2022_31717_MOESM1_ESM.pdf]

Supplementary Information

Freeze-derived heterogeneous structural color films

Shuangshuang Miao [a], Yu Wang [a], Lingyu Sun [a], Yuanjin Zhao[a,b,*]

[a] Department of Clinical Laboratory, Nanjing Drum Tower Hospital, School of Biological Science and Medical Engineering, Southeast University, Nanjing 210096, China

[b] Oujiang Laboratory (Zhejiang Lab for Regenerative Medicine, Vision and Brain Health); Wenzhou Institute, University of Chinese Academy of Sciences, Wenzhou, Zhejiang 325001, China

Email: yjzhao@seu.edu.cn

This supplementary file includes:

Supplementary Figs. 1 to 32

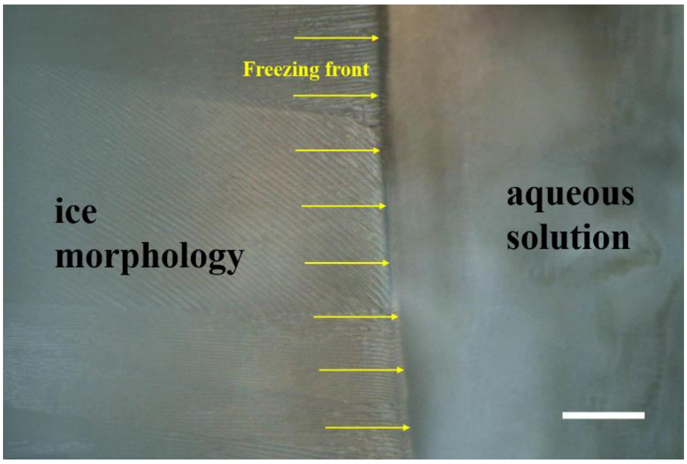

Supplementary Fig. 1 | The freezing process of sodium alginate solution (2.5 wt %). The scale bar is 500 μm.

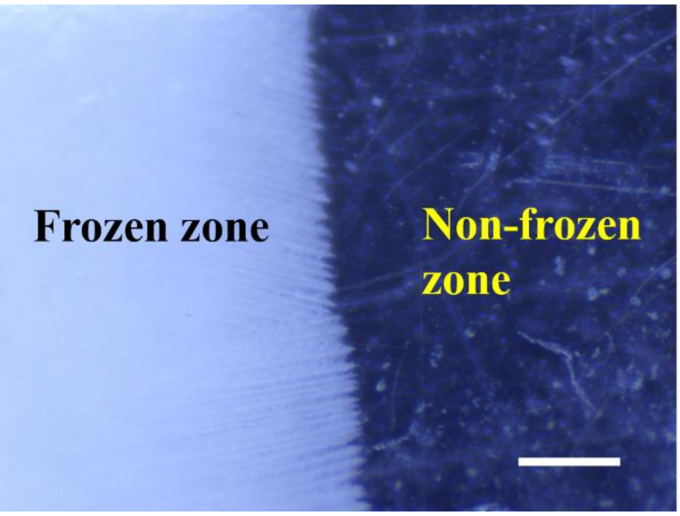

Supplementary Fig. 2 | The freezing front in PEGDA hydrogel fabricated by freeze-photopolymerization. The scale bar is 300 μm.

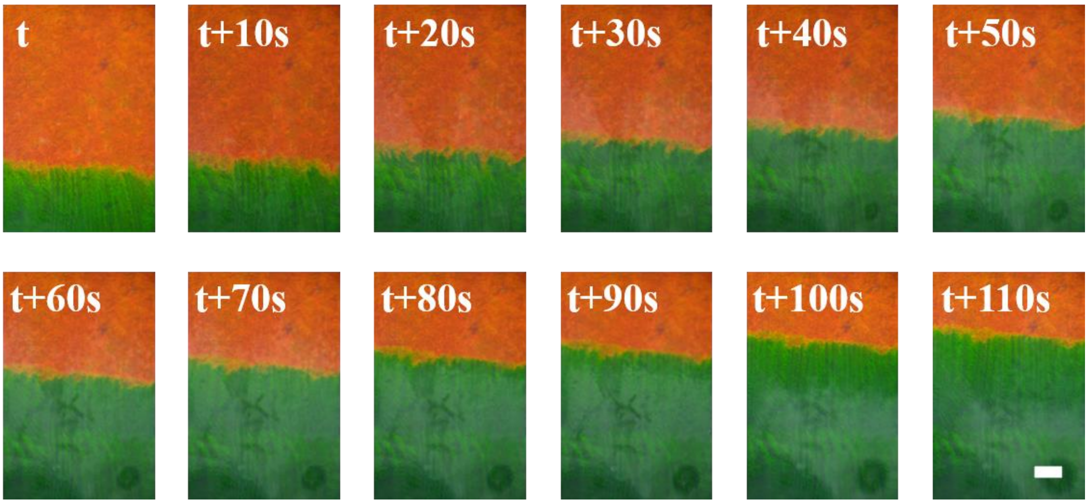

Supplementary Fig. 3 | The dynamic zoom-in images of freezing pregel suspension. Apparent color change and propelled ice crystals were simultaneous observed. The scale bars are 500 μm.

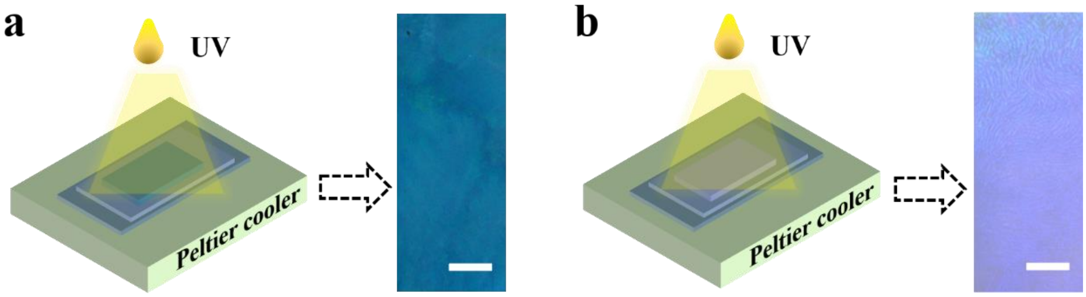

Supplementary Fig. 4 | The fabricated II-TSCHF with various colors. (a) The fabricated blue II-TSCHF. (b) The fabricated purple II-TSCHF. The scale bars are 500 μm.

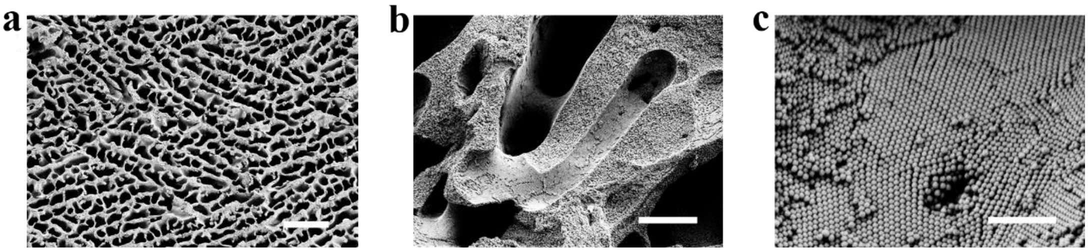

Supplementary Fig. 5 | SEM images of the surface of II-TSCHF. (a) SEM image of the surface of II-TSCHF. (b, c) Enlarged SEM images showing the micro-nano hierarchical structure. The scale bars are 40 μm in (a), 10 μm in (b), 2 μm in (c).

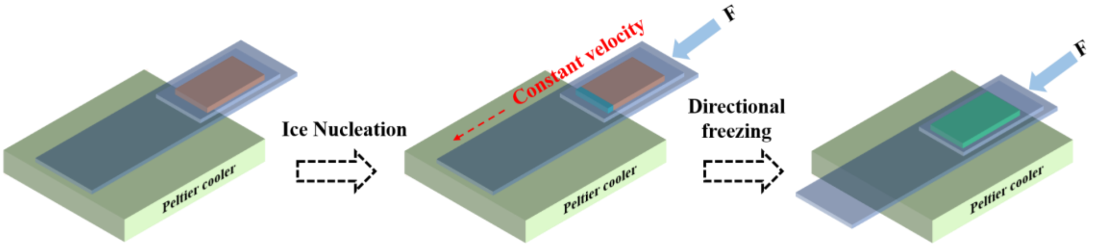

Supplementary Fig. 6 | Schematic illustration of directionally freezing pregel suspensions via constant velocity. In order to prevent the Hele-Shaw cell from being adhered to the Peltier substrate, ethanol was added to separate the glass slide and the Peltier cooler. Because the freezing point of alcohol is -117 °C and ethanol would remain liquid state during the moving process.

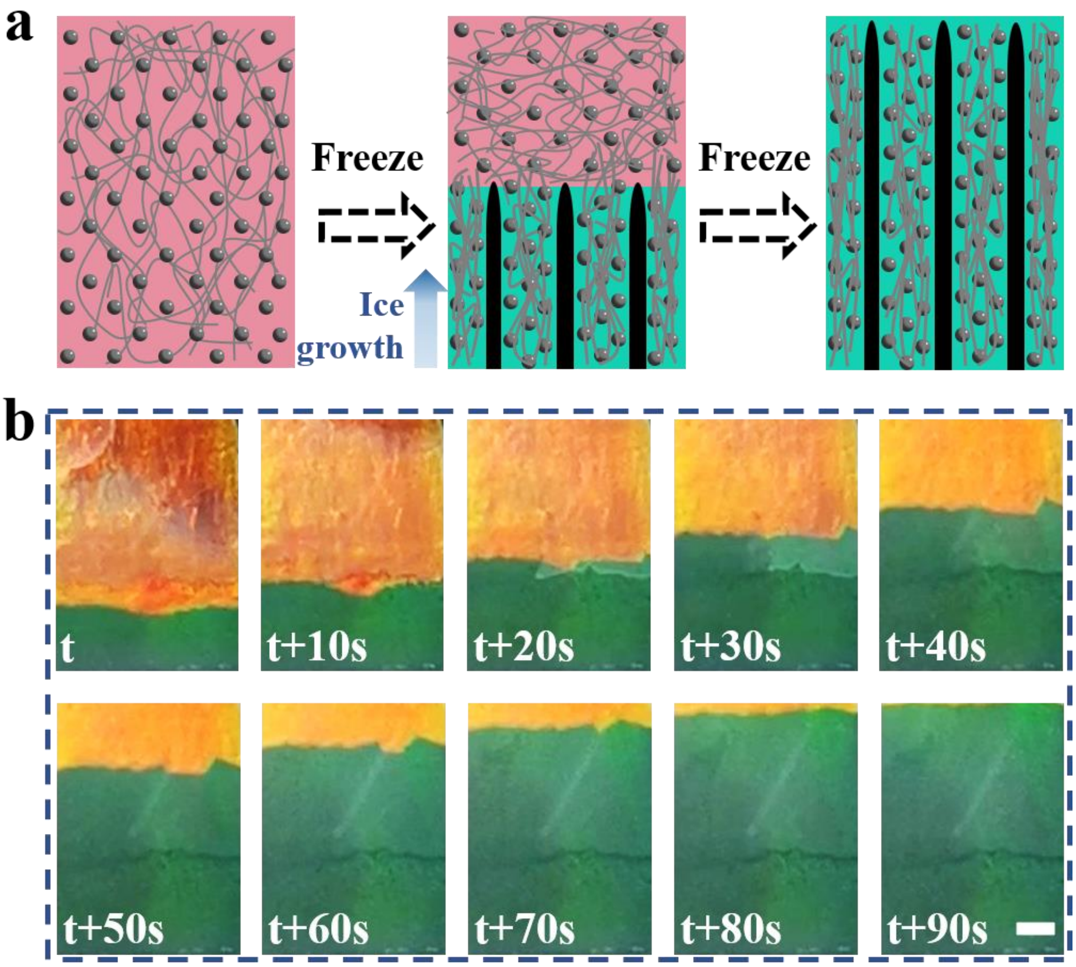

Supplementary Fig. 7 | The dynamic preparing process of directional II-TSCHF. The illustration (a) and experimental images (b) of directionally freezing pregel suspension. The scale bar is 1 mm.

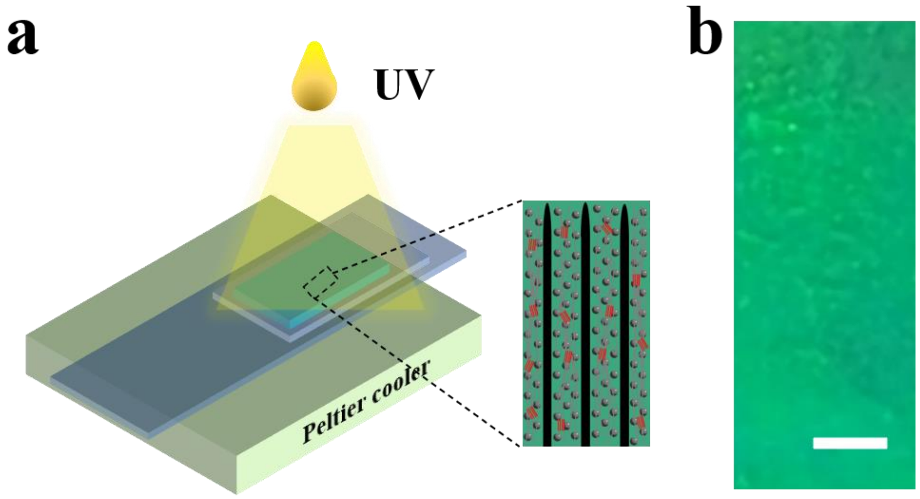

Supplementary Fig. 8 | The UV-solidification and resultant directional II-TSCHF. The schematic illustration (a) and experimental image (b) of the fabricated directional inverse ice-template structural color hydrogel film. The scale bar is 1 mm.

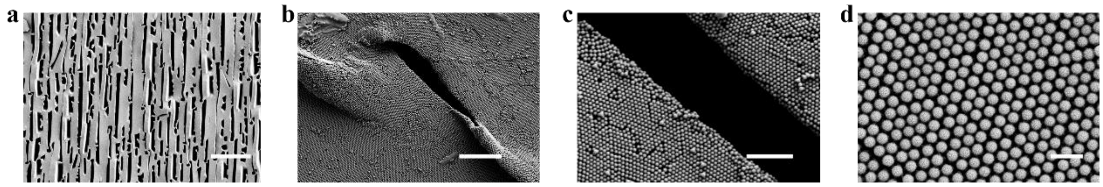

Supplementary Fig. 9 | SEM images of the surface of directional II-TSCHF. (a) SEM image of the surface of a directional II-TSCHF. (b-d) Enlarged SEM images showing the detailed structure. The scale bars are 100 μm in (a), 5 μm in (b), 2 μm in (c), 500 nm in (d).

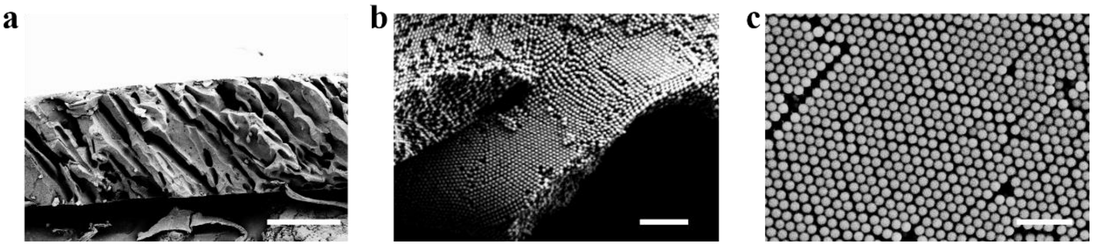

Supplementary Fig. 10 | SEM images of the cross section of directional II-TSCHF. (a) SEM image of the cross section of a directional II-TSCHF. (b, c) Enlarged SEM images showing the detailed structure.The scale bars are 100 μm in (a), 2 μm in (b), 1 μm in (c).

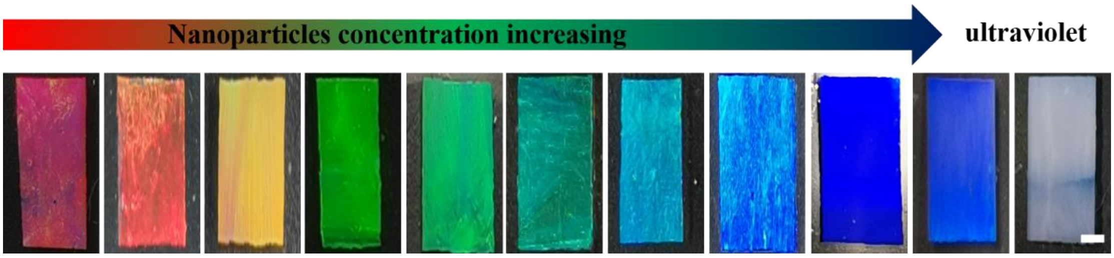

Supplementary Fig. 11 | The fabricated free standing II-TSCHFs with various colors. The scale bars are 2 mm.

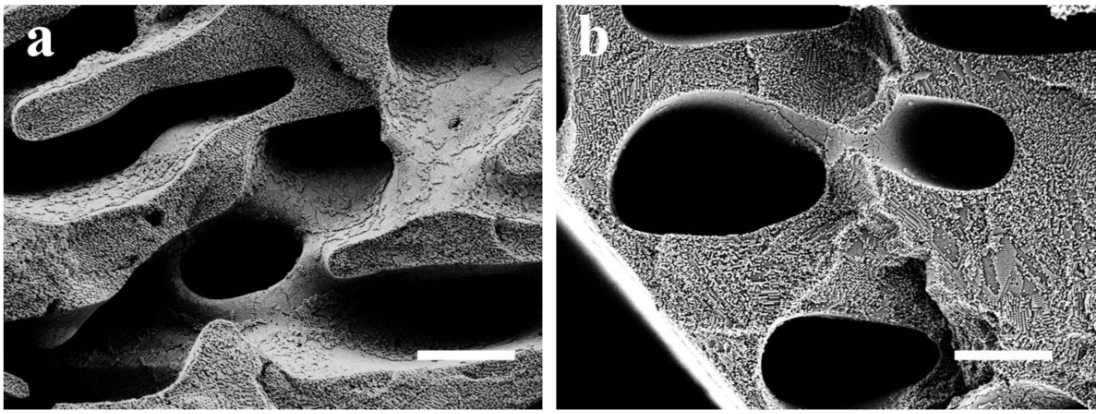

Supplementary Fig. 12 | Structure of two applicable ice-templated hydrogel materials. SEM images of the PAM II-TSCHF (a) and the PNIPAM II-TSCHF (b). The scale bars are 10 μm.

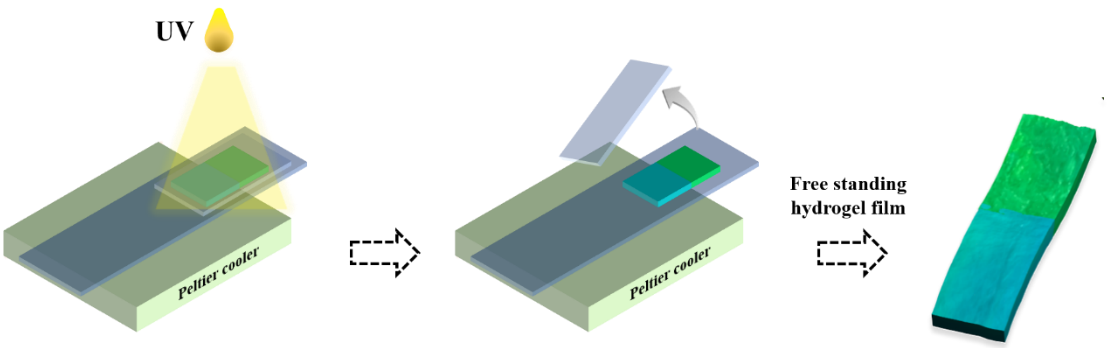

Supplementary Fig. 13 | Schematic illustration of fabricating heterogeneous hydrogel films with dual-color compartments.

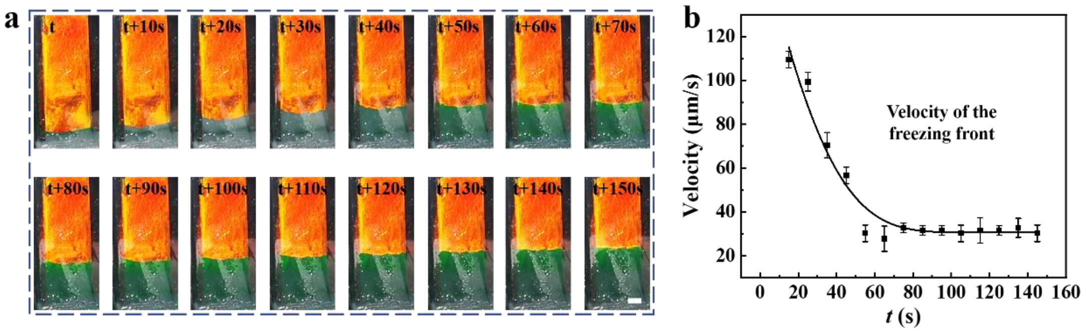

Supplementary Fig. 14 | The dynamic process of freezing pregel suspension. (a) The experimental images of fabricating dual-color hydrogel films. (b) Velocity of the freezing front changes with time. The error bars indicate the standard deviations of velocity derived from three locations of the freezing front. The scale bars are 2 mm.

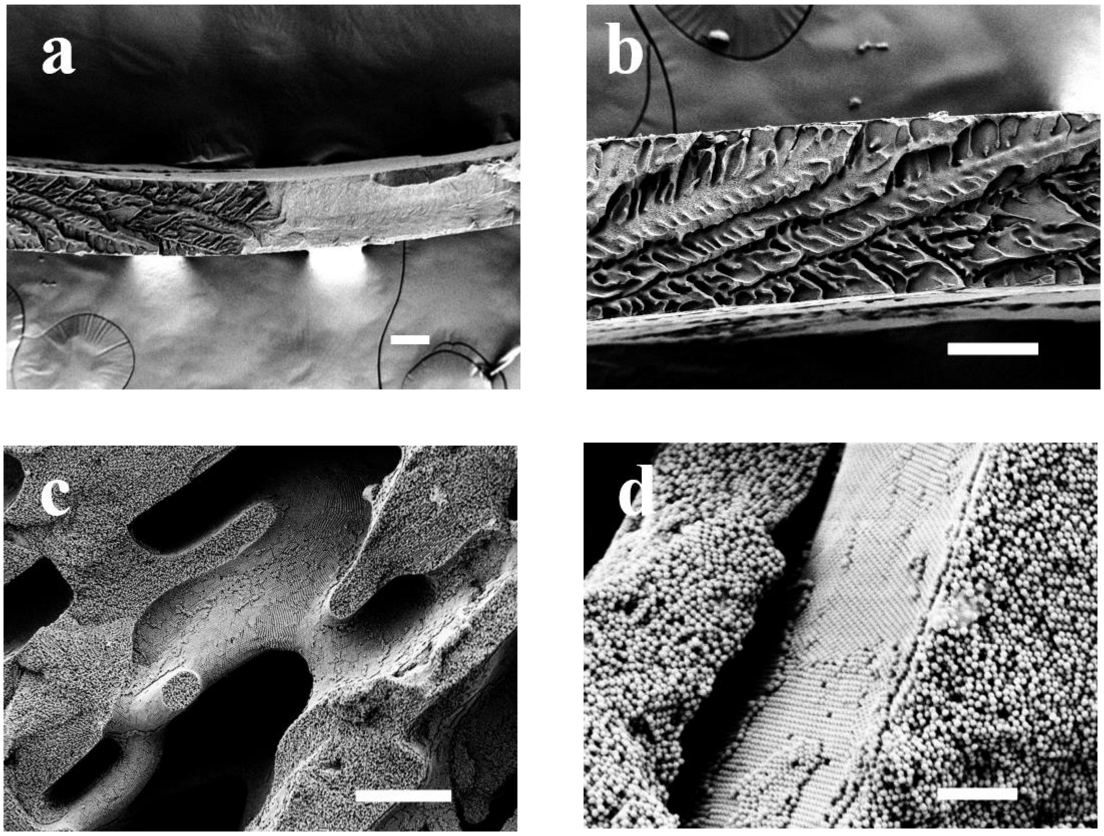

Supplementary Fig. 15 | SEM images of a dual color heterogeneous film corresponding to small subcooling. (a) SEM image of the cross section of a dual color heterogeneous film. (b-d) Enlarged SEM images showing the detailed structure. The freezing temperature was -6.9 °C. The v/v ratio of H_2O/PEGDA was 3:1. The diameter of nanoparticles was 172 nm. The scale bars are 100 μm in (a, b), 10 μm in (c), 2 μm in (d).

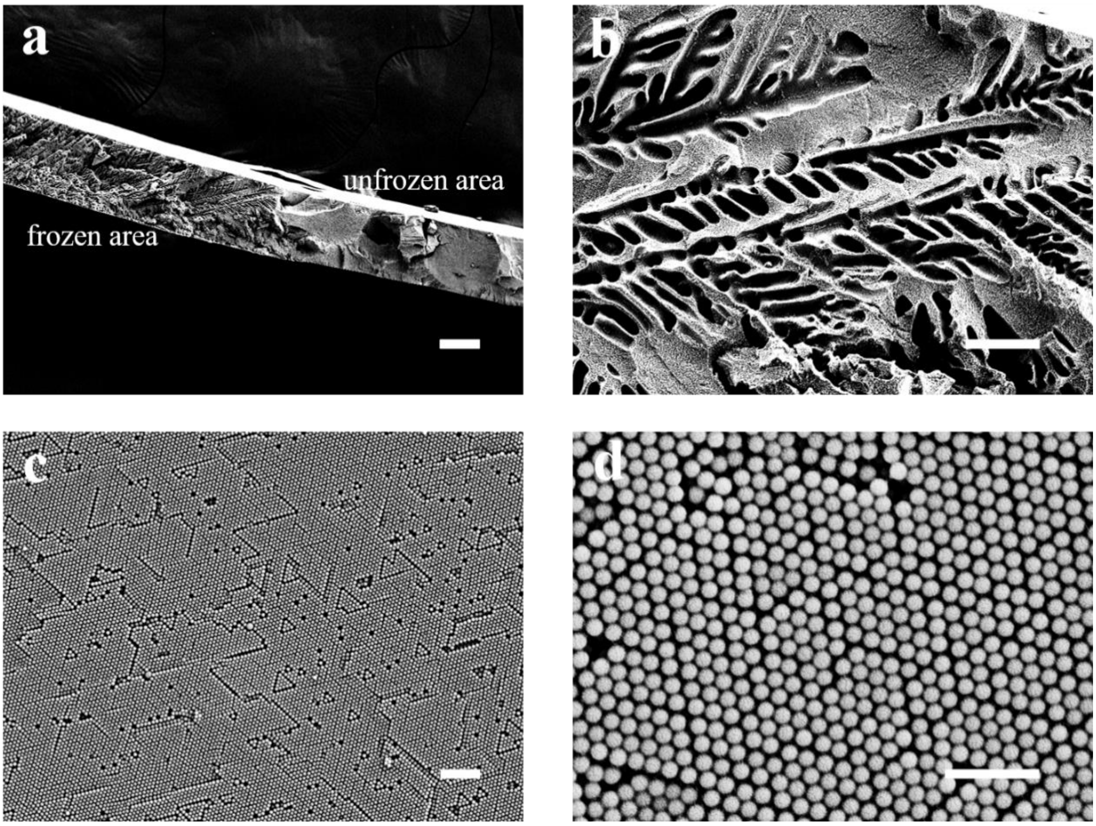

Supplementary Fig. 16 | SEM images of a dual color heterogeneous film corresponding to large subcooling. The freezing temperature was -12.3 ℃. The v/v ratio of H_2O/PEGDA was 3:1. Magnifying SEM image (b) of the cross section of frozen area in (a). Magnifying SEM image (c, d) of the cross section of non-frozen area. The diameter of nanoparticles was 172 nm. The scale bars are 100 μm in (a), 20 μm in (b), 2 μm in (c), 1 μm in (d).

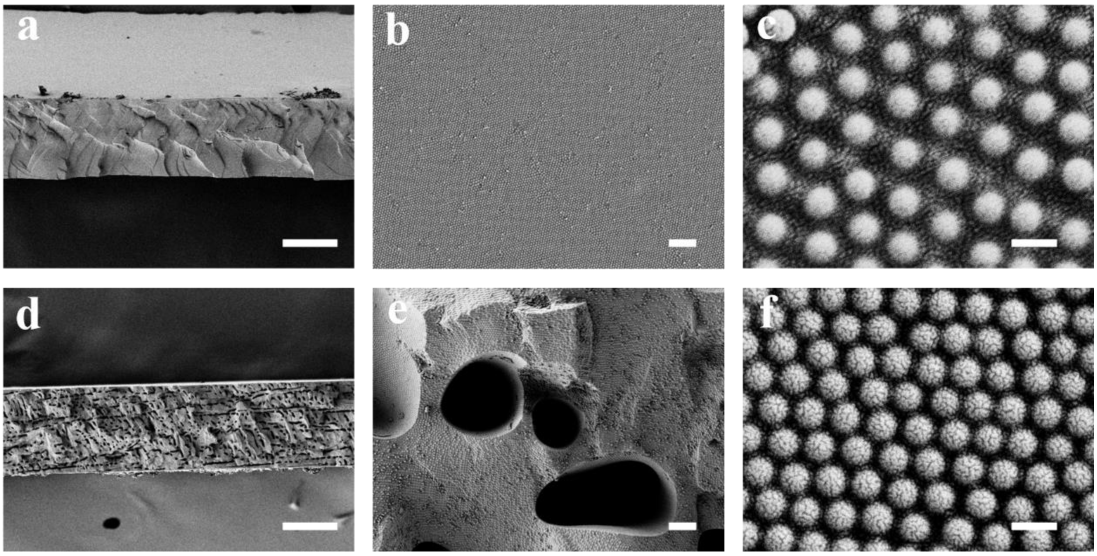

Supplementary Fig. 17 | Structural comparison of the frozen and non-frozen hydrogel films. Comparison of SEM images of structural color hydrogel without (a-c) and with (d-f) ice-templated structures. The freezing temperature is -5.9 ℃ for structural color hydrogel in (d-f). Scale bars are 100 μm in (a, d), 2 μm in (b, e), 200 nm in (c, f).

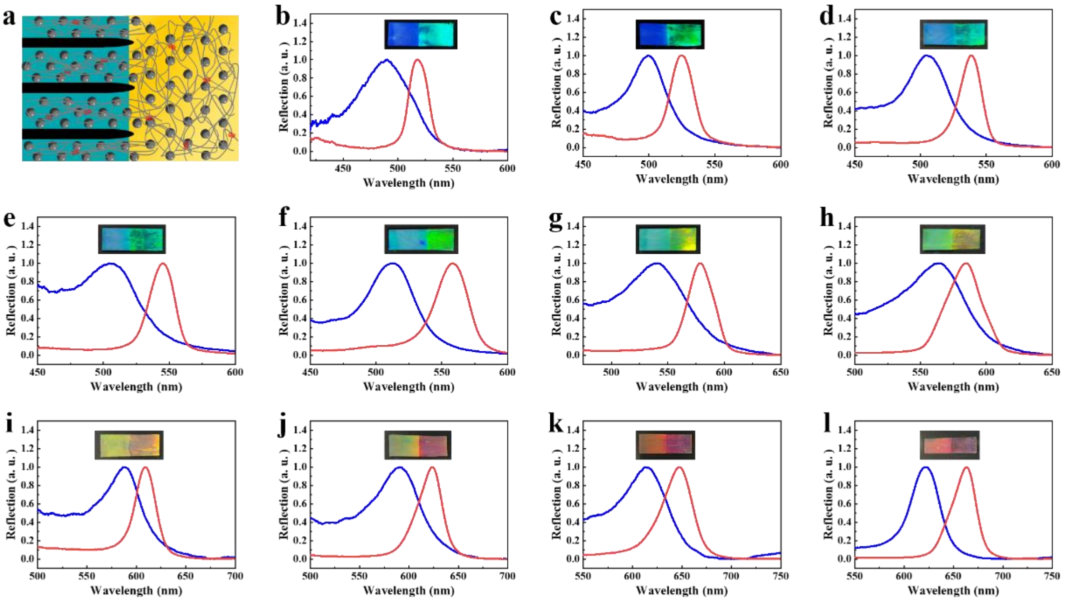

Supplementary Fig. 18 | The reflection spectra of various dual color films. (a) Schematic illustration of dual-color ice-template film. (b-l) The reflection spectra of various dual color films corresponding to Fig. 4d. The scale bars are 2 mm.

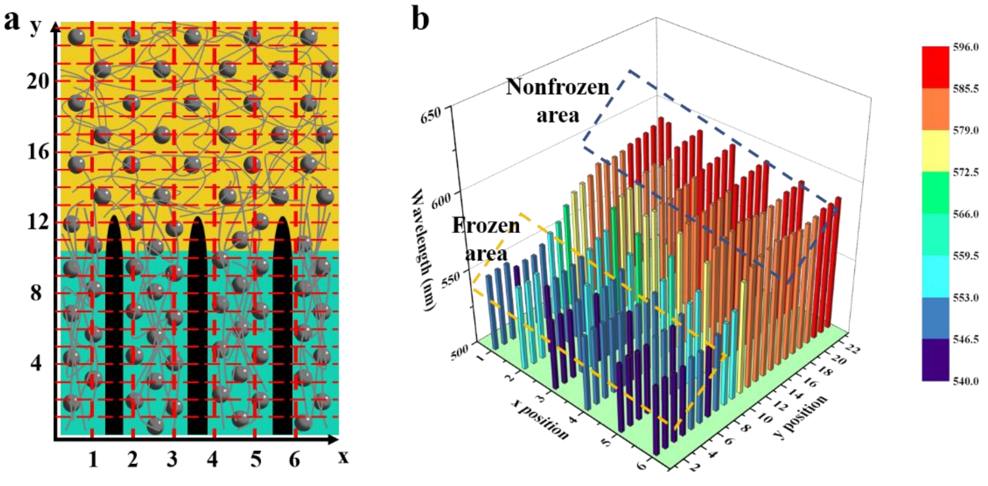

Supplementary Fig. 19 | The photonic band gap difference of the frozen and nonfrozen regions. (a) Schematic illustration of the positions of the array points on the dual-color heterogeneous structural color hydrogel films. (b) Spectral data of 138 array points (6 rows × 23 columns) on the surface of the dual-color heterogeneous films using spectrometer. The x and y coordinates are the position on the surface, and the z coordinate is the peak of the spectral data. The actual length represented by the unit length of the x-axis or y-axis is 0.6 mm. The subcooling is 7.2 K. The v/v ratio of H_2O/PEGDA was 1:1. Nanoparticles with a diameter of 145 nm were employed.

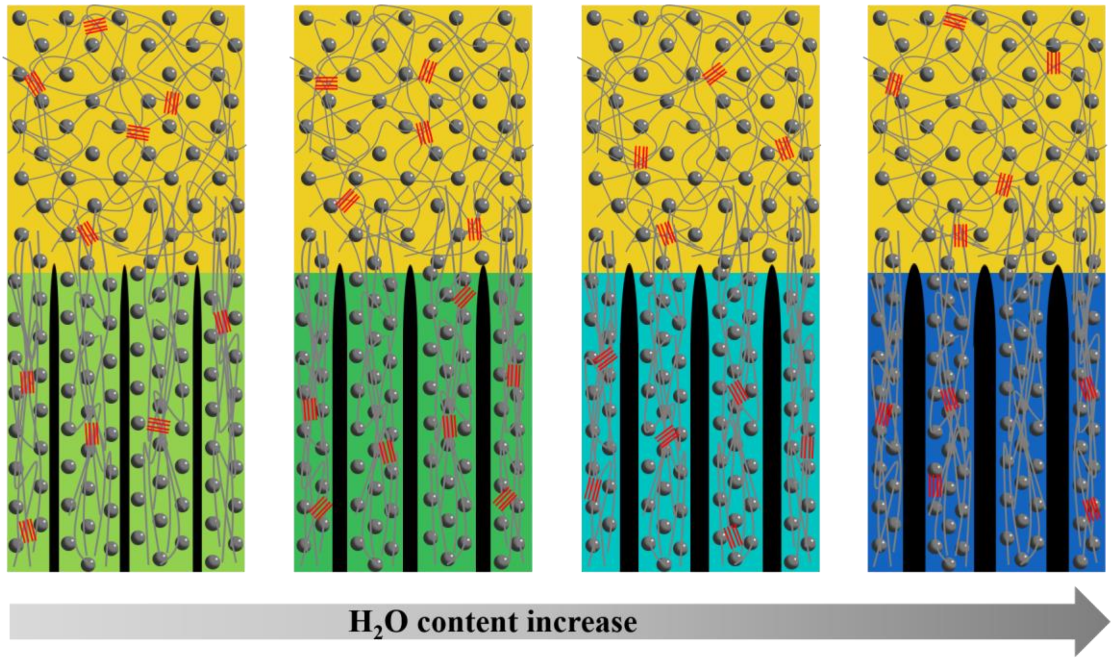

Supplementary Fig. 20 | The effects of H_2O content on the icing spectrum blue shift. Schematic illustration of the increasing blueshift value in the resulting hydrogels correspond to higher water content in pregel suspension.

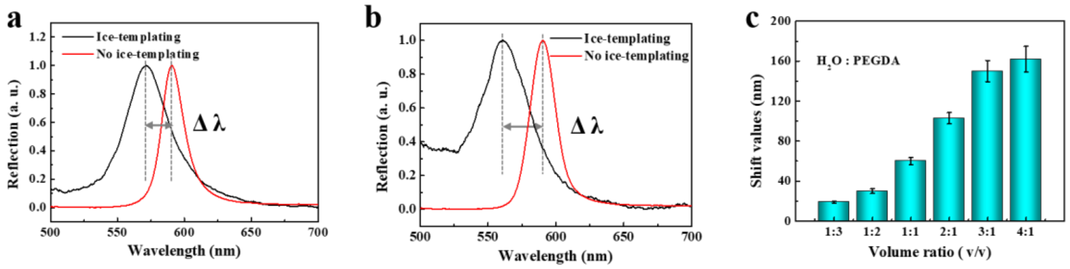

Supplementary Fig. 21 | The relations between blueshift values and the water content in pregels. (a, b) The wavelength shift when the volume ratio of H_2O: PEGDA is 1:3 in (a) and 1:2 in (b). (c) The function of blue shift varies with the water content in the PEGDA solution. The subcooling is 9.2 K. The error bars represent the standard deviations calculated from two specimens.

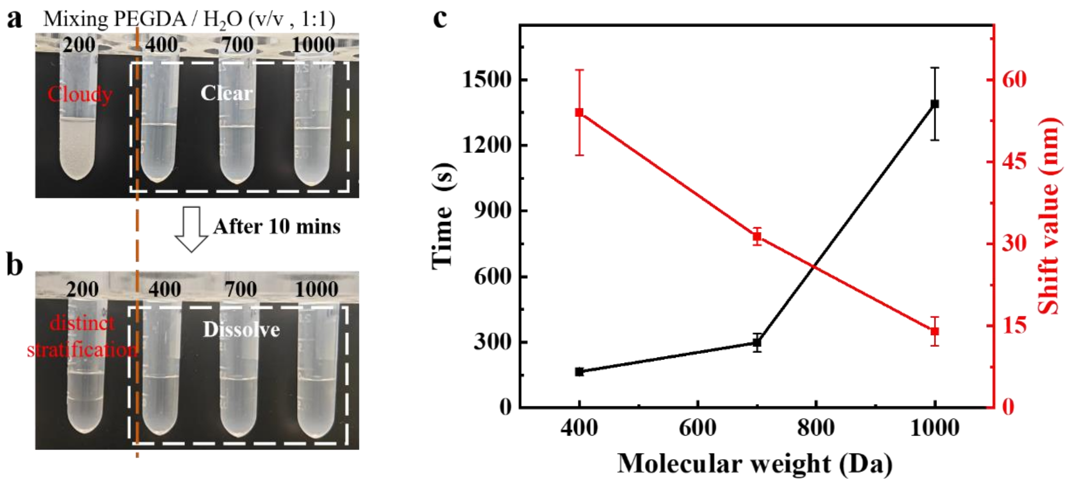

Supplementary Fig. 22 | The effect of molecular weight on freezing. (a, b) Optical images of mixing PEGDA (200, 400, 700, or 1000 Da) and H_2O at the v/v ratio of 1:1; (a) images was taken just after vigorously shaking with vortex; (b) images was taken after 10 mins. (c) The ice crystal growth time (black line) and shift value of wavelength blue shift (red line) change with average molecular weight variations of PEGDA under the same conditions. The ice crystal growth time is time from ice nucleation to the completely frozen state. The subcooling is 6.2 K. The v/v ratio of H_2O/PEGDA was 1:1. Nanoparticles with a diameter of 145 nm were employed. The error bars represent the standard deviations derived from three specimens.

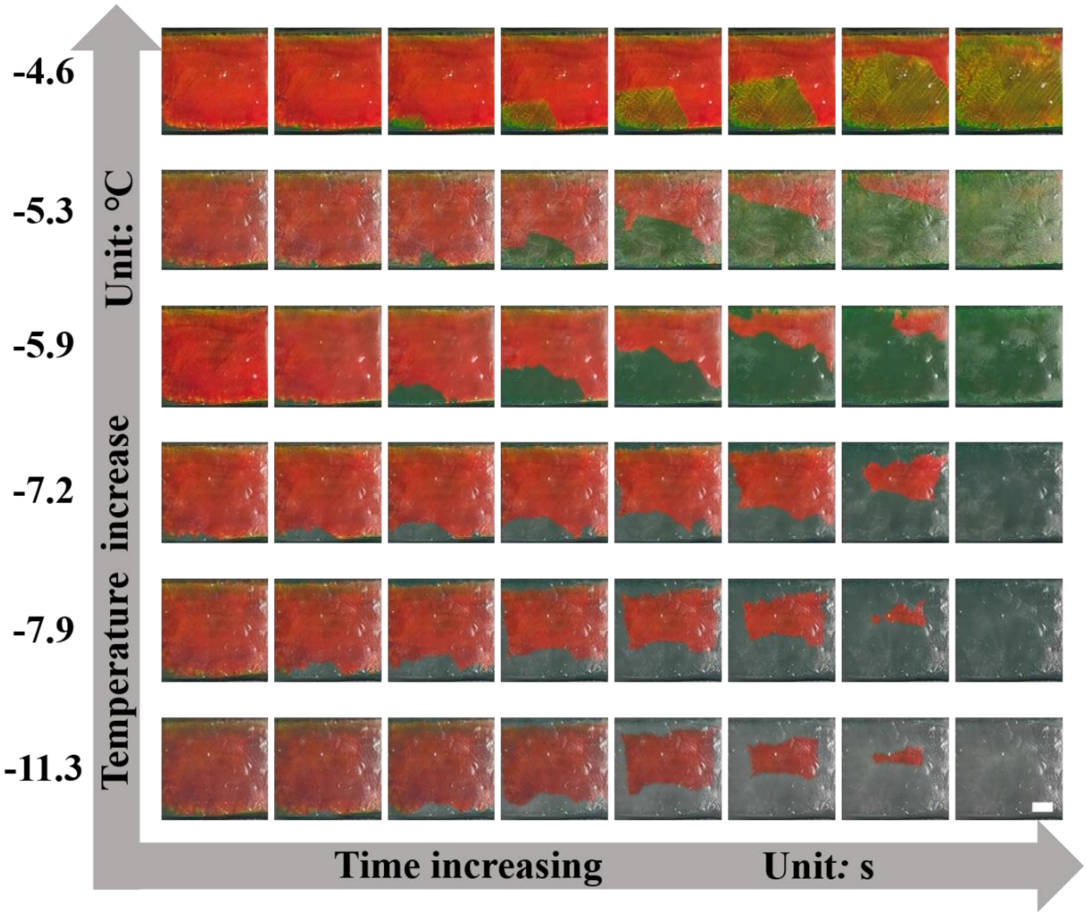

Supplementary Fig. 23 | The dynamic process of freezing pregel suspension on a uniform substrate under different freezing temperatures. The scale bar is 2 mm. The diameter of nanoparticles was 190 nm.

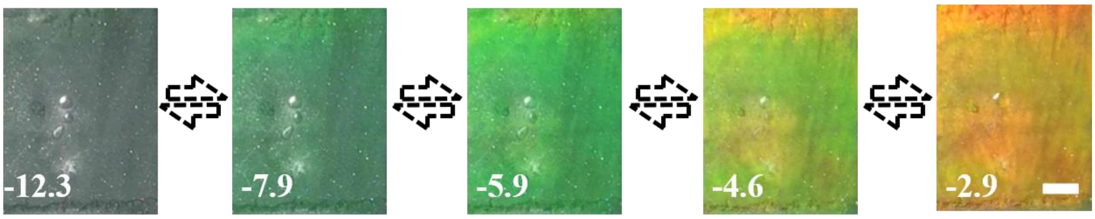

Supplementary Fig. 24 | Reversible conversions of optical band gaps of structural color pregels under two specified freezing temperatures. The scale bar is 2 mm. The diameter of nanoparticles was 145 nm.

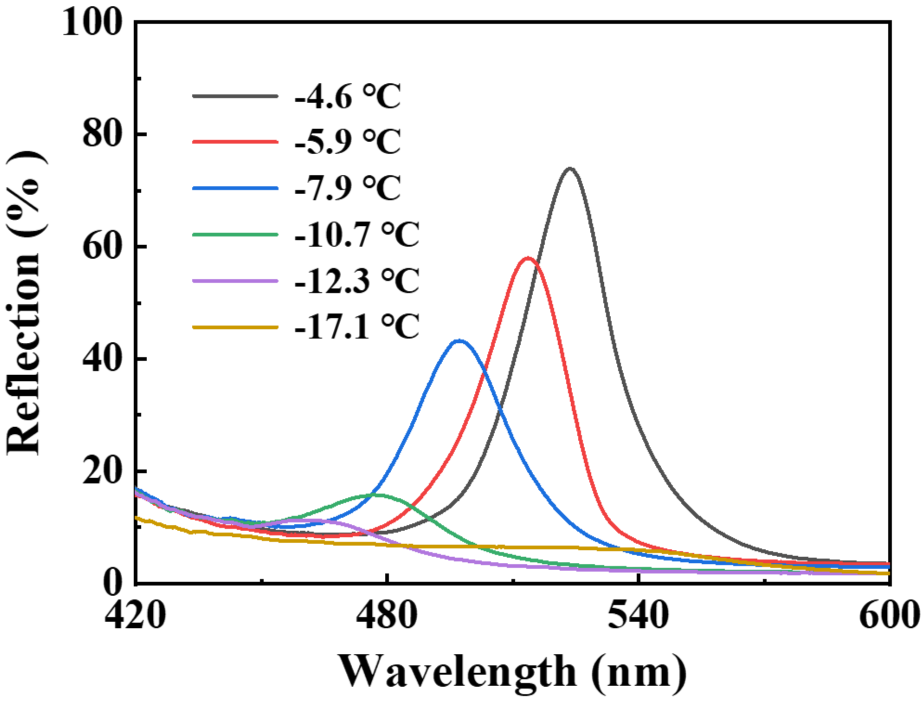

Supplementary Fig. 25 | The reflection spectrum under different Peltier temperatures.

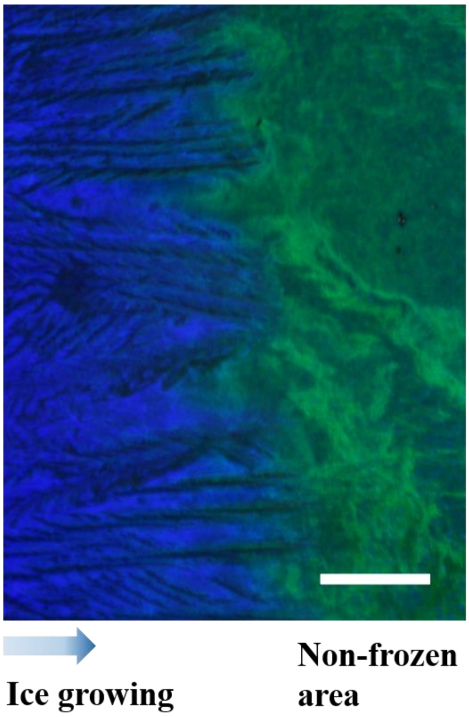

Supplementary Fig. 26 | Freezing front morphology. Optical microscopy images of the front of ice crystals, which shows the ice crystals' morphology at the solid-liquid interface of the II-TSCHFs. Distinguish structure and color can be seen between the frozen area and non-frozen area. The films were polymerized before the freezing front reached the boundary of the Peltier rectangular plate, which meant the freezing time was short. The scale bar is 500 μm.

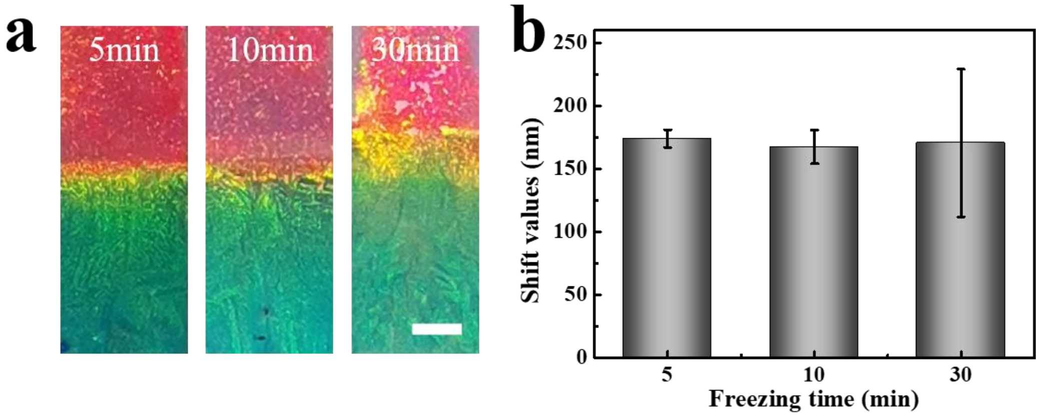

Supplementary Fig. 27 | The effects of freezing time on the icing spectrum blue shift of the heterogeneous hydrogel films. (a) Optical images of dual-color structural color films frozen at various freezing time. A transition zone visually appeared at the solid-liquid interface. And the transition zone widens over freezing time. (b) The wavelength shift values under various freezing time. The error bars represent the standard deviations calculated from three specimens. The scale bar is 2 mm.

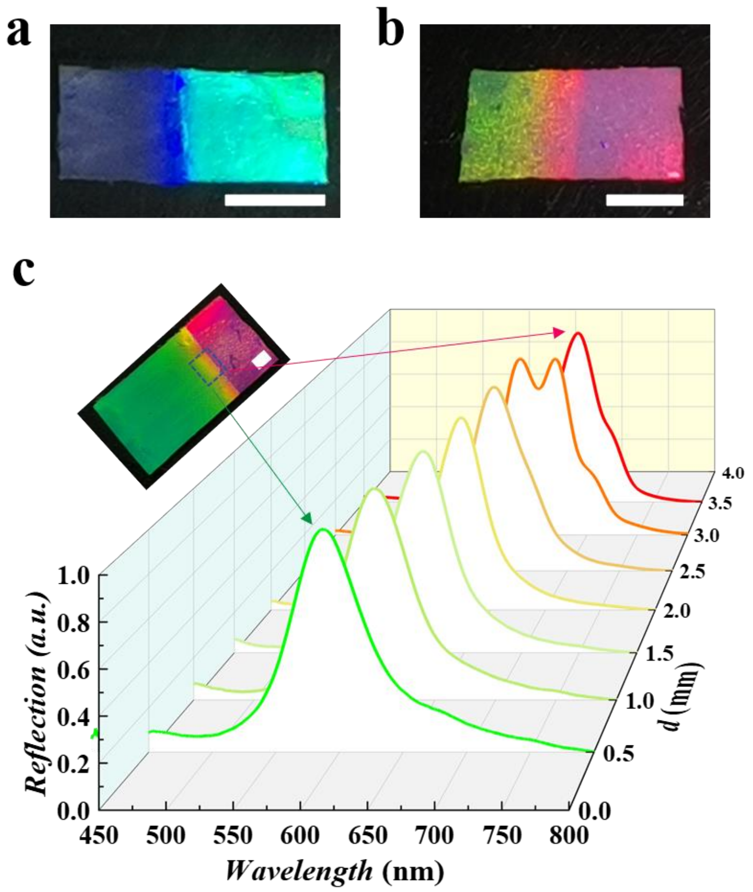

Supplementary Fig. 28 | Various dual-color structural color films with a transition zone at the freezing front. (a, b) Optical images of dual-color structural color films with a transition zone, including (a) blue transition zone and (b) orange transition zone. The films were polymerized after the freezing front reached the boundary of the Peltier rectangular plate for a long time, which meant the freezing time was long. (c) The spectrum of the transition zone of the two-component heterostructure structural color film. The location of the double peaks corresponded to the boundary of the ice crystals, which is the ice-water interface. The scale bars are 2 mm.

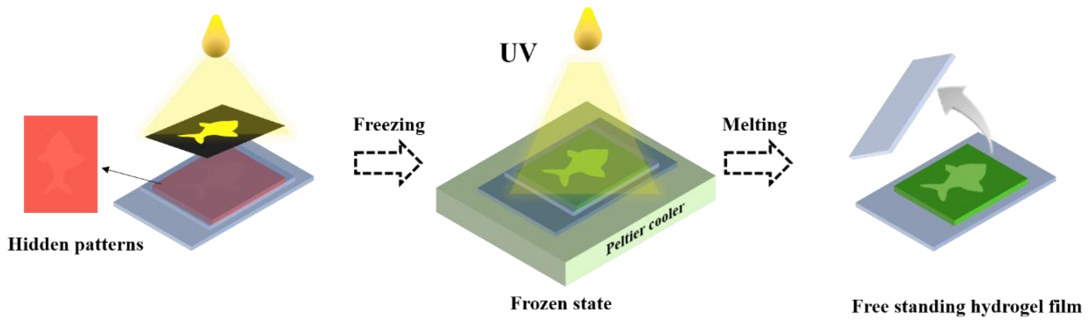

Supplementary Fig. 29 | Schematic illustration of patterning process on the II-TSCHFs. At room temperature, the suspension film was spatially irradiated via masks. Then the hidden patterns were displayed via freezing-photopolymerization method.

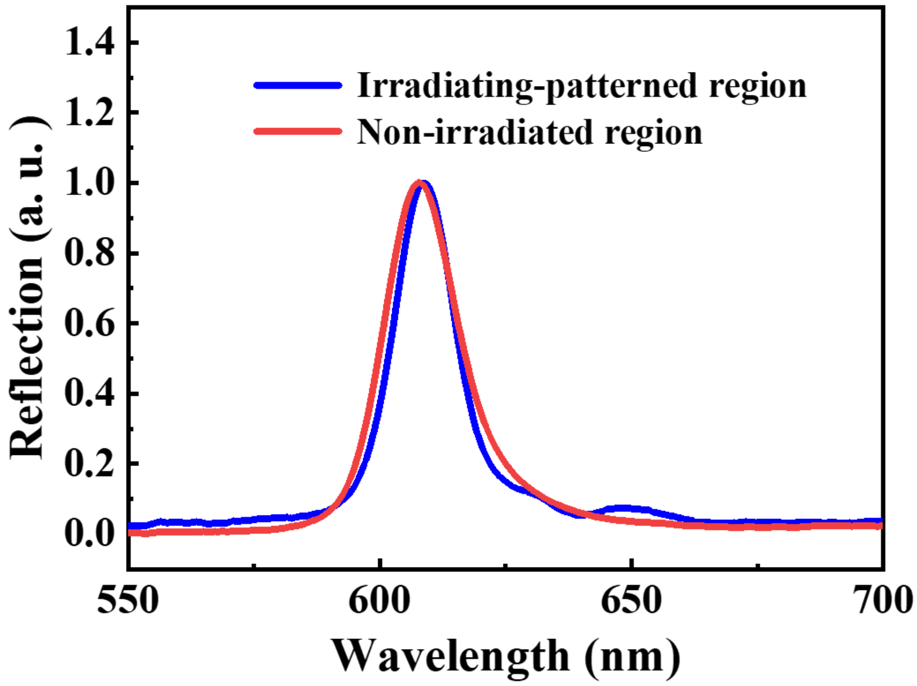

Supplementary Fig. 30 | The reflection spectra of the irradiating-patterned region and the non-irradiated region.

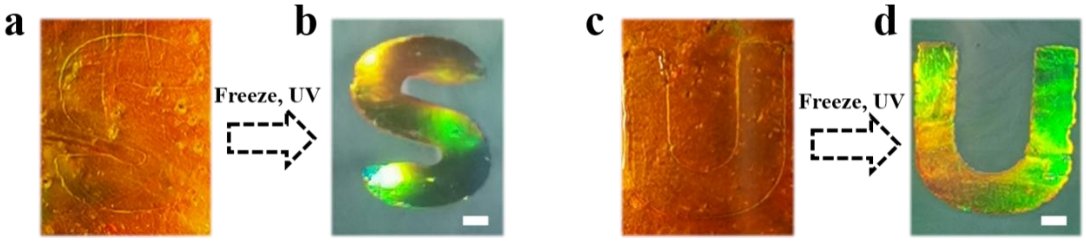

Supplementary Fig. 31 | Information encryption and decryption technology via icing wavelength blue shift. Optical images of structural color hydrogel with letter information, including the encrypted information (a, c) and the decrypted information (b, d). Scale bars are 1 mm.

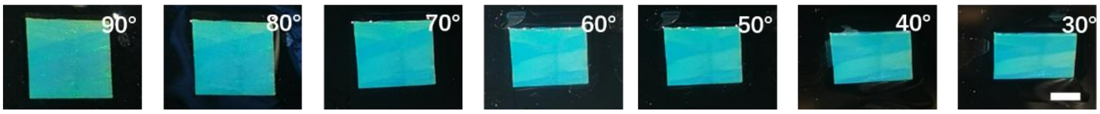

Supplementary Fig. 32 | Angle-dependance test for ice-templated structural color hydrogel films. Scale bar is 2 mm.
