# Supplementary material for: Freeze-derived heterogeneous structural color films
Source: Nat Commun. 2022 Jul 13;13:4044. doi: 10.1038/s41467-022-31717-2 (PMC9279407; doi:10.1038/s41467-022-31717-2)
Supplement: Supplementary file 2 — Description of Additional Supplementary Files [file 41467_2022_31717_MOESM2_ESM.pdf]

Description of Additional Supplementary Files

File Name: Supplementary Movie 1
Description: The zoom-in optical movie of ice crystals growth and icing wavelength blue shift.

File Name: Supplementary Movie 2
Description: The preparation of structural color films with dual colors via freeze-photopolymerization.

File Name: Supplementary Movie 3
Description: Structural color changes with temperature.

File Name: Supplementary Movie 4
Description: Five times freezing and melting cycles. The freezing temperature was -7.2 ℃.

File Name: Supplementary Movie 5
Description: The fabrication of heterogeneous structural color films with a fish pattern.

File Name: Supplementary Movie 6
Description: The freeze-decryption process of a fish pattern. The hidden pattern was displayed via freezing.
